# Sex and age at exposure influence ^131^I biodistribution and dosimetry in Sprague–Dawley rats

**DOI:** 10.1186/s13293-026-00989-4

**Published:** 2026-09-18

**Authors:** Anja Schroff, Klara Insulander Björk, Nishte Rassol, Julia Johansson, Torgny Lundberg, Hana Bakr, Martin Andersson, Johan Spetz, Eva Forssell-Aronsson

**Affiliations:** 1 Department of Medical Radiation Sciences, Institute of Clinical Sciences, Sahlgrenska Academy, University of Gothenburg, Sahlgrenska University Hospital, Gothenburg, SE-41345 Sweden; 2 Sahlgrenska Center for Cancer Research, Sahlgrenska Academy, University of Gothenburg, Gothenburg, SE-41390 Sweden; 3 Biomedical Engineering and Medical Physics, Sahlgrenska University Hospital, Gothenburg, SE-41345 Sweden

**Keywords:** Radioiodine, Thyroid, Absorbed dose, Sodium-iodide symporter, NIS

## Abstract

**Background:**

Assessing the biodistribution and absorbed dose of ¹³¹I is crucial for optimisation of radionuclide therapy, as well as for improving risk assessment following nuclear emergencies. After the Chernobyl accident, an increase in thyroid cancer among children was observed in regions affected by ¹³¹I fallout, particularly among young girls. Reasons for these age- and sex-specific differences in response to low doses of ¹³¹I are not fully understood, but variations in biodistribution and absorbed dose may be contributing factors. The aim of this study was therefore to assess the influence of sex and age at exposure on the biodistribution and dosimetry of ^131^I.

**Methods:**

Male and female Sprague Dawley rats were internally exposed to 0.36 MBq ¹³¹I at 5 or 17 weeks of age, corresponding to young and adult exposure groups. The ^131^I activity concentration of 16 vital tissues was measured at six time points (1–144 h post-injection) and used to estimate the mean absorbed dose to each organ. Sodium-iodide symporter (NIS) protein expression was assessed using immunohistochemistry and western blot.

**Results:**

The thyroid showed the highest ¹³¹I activity concentration at 18 h in males and 24 h in females, regardless of age. All non-thyroid tissues showed substantially lower activity concentrations, with the stomach having the second-highest levels. Statistically significant sex differences were determined in all tissues, with the most pronounced effect in the thyroid, where females consistently had higher activity concentrations compared to males. Age at exposure also impacted the biodistribution in some tissues. These differences led to pronounced variations in the absorbed dose. The absorbed dose to the thyroid ranged from 23 Gy/MBq in adult males to 100 Gy/MBq in young females. Protein quantification of NIS showed high individual variability but no clear difference between the groups.

**Conclusions:**

The results demonstrate that sex and age at exposure affect ¹³¹I biodistribution and absorbed dose in rats, particularly in the thyroid. The observed differences in absorbed dose may partly explain the variation in susceptibility to thyroid cancer in exposed populations reported after the Chernobyl accident. Further research is needed to clarify the underlying biological and mechanistic drivers of these differences.

**Supplementary Information:**

The online version contains supplementary material available at https://doi.org/10.1186/s13293-026-00989-4.

## Abstract (plain language summary)

Radioactive iodine (¹³¹I) is commonly used to treat thyroid diseases, including thyroid cancer. It can also be released during nuclear accidents, such as the Chernobyl accident in 1986. After the accident, thyroid cancer rates increased strongly in children exposed to radioactive iodine, especially in girls. However, it is still not fully understood why some groups are more affected by radioactive iodine than others. In this study, it was investigated whether sex and age influence how radioactive iodine is taken up and stored in the body. Male and female rats were used as a model organism and exposed to radioactive iodine at either a young or adult age. Afterwards, it was measured how much radioactive iodine was present in different organs over time and estimated how much radiation dose these organs received. The thyroid gland showed the highest uptake of radioactive iodine in all groups. Female rats stored radioactive iodine in the thyroid for a longer time than males, which resulted in a higher radiation dose to the thyroid. Young males also showed higher thyroid exposure than adult males. Other organs generally showed much lower levels of radioactive iodine. These findings show that sex and age can strongly affect how radioactive iodine behaves in the body, especially in the thyroid gland. This may help explain why some populations are more sensitive to radiation-related thyroid disease. The results may also be important for improving radioactive iodine treatment and for estimating radiation risk after nuclear accidents and radioactive iodine exposure.

**Supplementary Information:**

The online version contains supplementary material available at https://doi.org/10.1186/s13293-026-00989-4.

## Highlights

Sex and age at exposure significantly influenced the biodistribution and dosimetry of ¹³¹I in rats.

Female rats exhibited higher and prolonged thyroid uptake of ¹³¹I, resulting in substantially higher absorbed doses to the thyroid compared with males, regardless of age.

Age-related differences in thyroid uptake and absorbed dose were primarily observed in male rats, with young males showing high ¹³¹I uptake leading to a higher absorbed dose than adult males.

The thyroid showed the highest ¹³¹I uptake in all groups, whereas all non-thyroid tissues exhibited substantially lower activity concentrations and absorbed doses.

No consistent differences in total NIS protein expression were observed between groups, suggesting that factors beyond NIS abundance may contribute to sex-dependent differences in thyroid iodine uptake.

**Supplementary Information:**

The online version contains supplementary material available at https://doi.org/10.1186/s13293-026-00989-4.

## Introduction

The radioactive iodine isotope ^131^I was among the first radionuclides used in nuclear medicine and remains in frequent use today for the treatment of hyperthyroidism and differentiated thyroid cancer [1]. This is because iodine naturally accumulates in the thyroid gland, where it is used for production of the iodine-containing thyroglobulin, a thyroid hormone precursor. From there the prohormone thyroxine (T4) is synthesised, secreted from the gland, and converted in the tissue to the active hormone triiodothyronine (T3), which is involved in the regulation of the body’s metabolism [2]. Iodine uptake into thyroid follicular cells is facilitated by the sodium-iodide symporter (NIS), a plasma membrane glycoprotein that transports 2 sodium ions for each iodide ion into the cells [3]. In addition to treatment of thyroid disorders, ^131^I can be used as a radiolabel for pharmaceuticals targeting non-thyroid diseases, and for diagnostic, pre- and post-therapy imaging by single-photon emission computed tomography (SPECT). ^131^I undergoes β^−^ decay, with emission of electrons with an energy of 606 keV (89% abundance) and a mean energy of 192 keV, as well as a principal photon of 364 keV (82% abundance), and has a half-life of about 8 days.

Other iodine isotopes, such as ^123^I, ^124^I and ^125^I, are also widely used in nuclear medicine and research. ^123^I is sometimes the preferred isotope for diagnostic SPECT imaging, while ^124^I is primarily used for positron emission tomography (PET) imaging, and ^125^I can be used for SPECT imaging but is more frequently used as a radiolabel in research applications. Although all four isotopes have different radioactive decay characteristics, they share similar chemical properties and therefore share the same physiological biodistribution and biological behaviour.

Apart from its important medical applications, ^131^I is also a byproduct of nuclear fission and one of the most concerning radionuclides released following a nuclear emergency or the detonation of nuclear weapons. The Chernobyl accident in 1986 was one of the biggest nuclear emergencies in history, and in the months following the event large areas were affected by ^131^I fallout. Radioactive ^131^I was deposited onto pasture vegetation, where it was ingested by grazing dairy cattle and subsequently accumulated in their milk. This resulted in substantial thyroid exposure, when locally produced contaminated dairy products were consumed. In subsequent years, an increase in paediatric thyroid cancer was observed in the most heavily fallout-affected regions, likely associated with exposure to ingested ^131^I [4–7]. This increase was more pronounced in girls than in boys, whereas no corresponding increase was observed in adults, regardless of sex [8–10]. These epidemiological observations suggest that age and sex influence the susceptibility to ^131^I-induced thyroid cancer.

One possible explanation for these differences is variation in iodine uptake and retention in the thyroid, which directly affects the exposure. Higher uptake or prolonged retention of ^131^I in the thyroid would result in a higher absorbed dose and may potentially increase the risk of cancer development. Since iodine uptake is mediated by NIS, differences in NIS expression or function between sexes and at different ages could contribute to differences in iodine accumulation and, consequently, the absorbed dose. However, it remains unclear to what extent age- and sex-dependent differences in iodine biokinetics influence absorbed dose.

Accurate assessment of the biodistribution and biokinetics of ^131^I is therefore important for assessing the absorbed dose to the thyroid and other organs. This is essential for identifying individuals at risk following accidental and deliberate exposure and providing appropriate treatment and health care.

To study the biokinetics of ^131^I as a function of age at exposure and sex, controlled in vivo studies in rodents are necessary. Mice and rats are important in vivo models used to test new radiopharmaceuticals or optimise the use of existing drugs, and they allow investigation of confounding factors such as sex and age that would not be possible in humans. The larger size of rats compared to mice is advantageous, since sampling of especially small organs, such as the thyroid, is easier and more tissue is available for further analyses.

We previously demonstrated that the biodistribution of ^131^I is affected by circadian variation in 9- to 10-week-old mice and that it differs from that of other radiohalogens in young male rats [11, 12]. Other studies have also investigated thyroid uptake and biodistribution in mice and rats of different strains, ages, and sexes, as well as the impact of diurnal variation [13–19]. However, most studies reported the biokinetics of ^131^I for only one age group (adult or young rodents) or one sex (male or female). The impact of age at exposure on the biodistribution of ^131^I in rats, as a frequently used model organism, is therefore unknown, and investigations of direct sex differences in a single study are rare, as only one sex is often examined.

The purpose of this study was therefore to compare the biodistribution of ^131^I in male and female rats of two different age cohorts (5 and 17 weeks) and to determine the absorbed dose of ^131^I to a selection of important organs and tissues. In this way, the uptake of ^131^I was studied as a function of age at exposure and sex. In addition, NIS protein expression in the thyroid was assessed to explore whether differences in iodine uptake are associated with sex- and age-dependent variation in iodide transport. Together, these approaches were used to investigate whether differences in iodine uptake and absorbed dose may contribute to variation in response to ^131^I exposure.

## Materials and methods

### Animals

Sprague-Dawley rats (Janvier Labs, Le Genest-Saint-Isle, France) of both sexes were exposed at 5 weeks (young cohort) and at 17 weeks (adult cohort). The average body weight by age and sex are provided in Table 1. Animals were housed in standard rat cages with free access to standard rat chow (6 mg/kg stable iodine) and water. One week before exposure, rats were switched to a diet with lower stable iodine content (1 mg/kg stable iodine; LabDiet^®^ 5001, IPS Product Supplies, London, UK), which was maintained throughout the study.

**Table 1:** Characteristics of the different study groups

	Body weight [g]	Photon $\:\phi\:$ for whole body	Electron $\:\phi\:$ for thyroid
Male, Young	210 (2)	0.099	0.69
Female, Young	170 (1)	0.099	0.68
Male, Adult	620 (4)	0.13	0.70
Female, Adult	330 (4)	0.11	0.69

Average body weight [is presented as mean (SEM) (*n* = 50)] assessed from similar rats in a follow-up study. The self-absorbed fraction, ϕ, for whole body from photons was based on data from Stabin and Konijnenberg (2000) [20]. The self-absorbed fraction for thyroid from electrons was based on the data from Endo et al. (1998) [21].

### Radionuclide preparation

^131^I was received as sodium iodide solution (GE HealthCare, Danderyd, Sweden) or in the form of sodium iodide (^131^I) capsules (Curium Pharma, Stockholm, Sweden). Capsules were dissolved in 5 mL saline (Braun, Melsungen, Germany).

The activity of the ^131^I stock solution was measured using an ionisation chamber (CRC^®^-15R, Capintec Inc., Florham Park, New Jersey, USA).

### Exposure and tissue sampling

For each injection day, a stock solution of ^131^I was prepared by diluting the batch solution with saline to the desired activity concentration. Syringes were prepared, individually weighed before and after injection, to determine the injected volume, and measured in the ionisation chamber to account for variation between syringes.

For each age and sex group, 30 rats (36 for adult males) were randomly assigned to six groups of five animals each (six for adult males). Rats were intravenously injected via the tail vein with an average of 0.36 MBq (SEM = 0.005) ^131^I dissolved in approximately 0.2 mL. If leakage occurred at the injection site, it was collected using a compress and retained for subsequent activity measurement. The injected ^131^I activity for each rat *A*_*inj*_ was determined according to:

1 $$\:{A}_{inj}={A}_{before}-{A}_{after}-{A}_{compress},$$

where *A*_*before*_ represents the activity of the syringe before injection measured in the ionisation chamber, *A*_*after*_ the activity of the syringe after injection measured in an automated gamma counter (2480 WIZARD2^®^, PerkinElmer, Waltham, Massachusetts, USA), and *A*_*compress*_ the activity of the compress, also measured in the gamma counter. The ionisation chamber and the gamma counter were cross-calibrated (see details below).

Rats were euthanised at 1, 6, 18, 24, 72, or 144 h post-injection by cardiac puncture under anaesthesia with Allfatal vet. (100 mg/ml sodium pentobarbital; Omnidea AB, Stockholm, Sweden), followed by dissection. One thyroid lobe, blood (~ 1 mL, obtained from cardiac puncture), bone marrow from one femur, salivary gland, neck muscle, heart, lung, brain, liver, one kidney, spleen, as well as emptied stomach and emptied intestines (small and large), were sampled. In addition, both ovaries were collected from female rats and both testes from male rats at all time points for the young cohort, but only at 6, 24, and 144 h for the adult groups. At these times, the remaining carcasses were also retained. Carcass was defined as all remaining tissues not collected separately for individual analysis. The remaining thyroid lobe was flash-frozen in liquid nitrogen and preserved for future analyses.

Tissues contaminated with blood or gastrointestinal content were rinsed in saline. All tissues were placed in pre-weighed scintillation vials, which were weighed again with the organ inside to determine organ mass. To enable further analyses, 4% buffered formaldehyde (HistoLab, Askim, Sweden) was added to all solid samples after weighing.

It should be noted that not all rats in the 1-h group were euthanised exactly 60 min post-injection. The exact time of death for each animal was used in the biokinetic modelling and dose estimations.

### Activity measurement in tissues

The activity in the collected organs was measured using the gamma counter. The gamma counter was calibrated for ^131^I, and the measurement window was set to 320–410 keV. The measurement time was adjusted to ensure that the number of counts exceeded 1000 above background, corresponding to a detection error of less than 3%. All activity measurements were corrected for background. The detector dead time was determined and found to be sufficiently low for the activity range investigated, so no dead-time correction was applied.

To convert measured counts to activity in Bq, a dilution series was performed to generate a calibration curve, and the gamma counter was cross-calibrated against the ionisation chamber. Additionally, potential volume effects were assessed and found to be negligible. Due to the larger size of the rat carcasses, the ^131^I activity in these samples was measured in the ionisation chamber and corrected for background.

### Activity concentration in tissues

For the various time-points, the activity concentration *c*_*tissue*_*(t)* was calculated for each tissue, expressed as the percentage of injected activity per gram of tissue [%IA/g]

2 $$\:{c}_{tissue}\left(t\right)=\frac{{A}_{tissue}\left(t\right)}{{A}_{inj}*{m}_{tissue}}*100\:,$$

where $\:{A}_{tissue}\left(t\right)$ is the measured activity in the tissue sample at time * t*, decay corrected to the time of injection$\:({t}_{0})$ to remove the physical decay of ^131^I, $\:{A}_{inj}\:$is the injected activity at $\:{t}_{0}$, and $\:{m}_{tissue}$ is the mass of the tissue.

### Biokinetic modelling

Biokinetic modelling was performed for each tissue using the previously calculated activity concentrations, decay corrected to the time of euthanasia. The average activity concentration [%IA/g] was plotted against time after injection in Excel (Microsoft Corporation, Redmond, Washington, USA), and curve fitting was performed using the least-squares method via the Solver plug-in. A mono-exponential curve was first fitted to the last three time points, representing the slow phase of activity reduction. This curve was then back-extrapolated to the earlier three time-points, and the calculated values were subtracted from the corresponding measured data points. A second mono-exponential curve was subsequently fitted to these adjusted data points to represent the fast phase of activity reduction. The sum of the two mono-exponential curves was considered the best fit for each dataset. For the thyroid, where a pronounced uptake phase was observed with a peak between 18 and 24 h, biexponential curve fitting was performed starting from the time point with the maximum activity concentration.

### Time-integrated activity concentration estimation

The time-integrated activity, $\tilde A({r_S},{T_D})$, in each source region, $\:{r}_{S},$ was defined as the integral of the activity in $\:{r}_{S}$ from the time of injection$\:({t}_{0})$ to the dose determination time $\:{T}_{D}$, representing the study endpoints, as well as infinity.

3 $$\tilde A({r}_{S},{T}_{D})=\:{\int}_{{t}_{0}}^{{T}_{D}}A\left({r}_{s},t\right)dt,$$

Since the biokinetic curves in the present study were fitted to activity concentrations, the corresponding time-integrated activity concentration, $\:{\stackrel{\sim}{c}}_{tissue}\left({T}_{D}\right)$, was obtained by integration of the fitted activity-concentration curves.

4 $$\:{\stackrel{\sim}{c}}_{tissue}\left({T}_{D}\right)=\:{\int}_{{t}_{0}}^{{T}_{D}}{c}_{tissue}\left(t\right)dt=\:\frac{\tilde A({r}_{S},{T}_{D})}{{A}_{inj}*{m}_{tissue}}*100,$$

The activity at $\:{t}_{0}$ was assumed to be zero for all organs except blood. For blood, the entire injected activity was assumed to be present in the blood at $\:{t}_{0}$, and the initial activity concentration was therefore calculated from the estimated total blood mass. Total blood volume in mL, was determined for each study group according to Lee and Blaufox (1985) [22]

5 $$\:{V}_{blood}=0.06*{m}_{body}+0.77,$$

where $\:{m}_{body}$ is the group-average body weight presented in Table 1. Total blood mass, $\:{m}_{blood}$, was calculated from $\:{V}_{blood}$ assuming a blood density of 1.06 g/mL. The initial blood activity concentration was then calculated using Eq. (2).

The contribution from $\:{t}_{0}$ to the first measurement point was calculated using the trapezoidal method for all tissues. For the thyroid, the trapezoidal method was applied from $\:{t}_{0}$ to the time of maximum activity (18–24 h), followed by integration of the biexponential curve.

### Absorbed dose estimations using an analytical method

The absorbed dose of ^131^I to each tissue was calculated as a function of time according to the Medical Internal Radiation Dose (MIRD) formalism [23],

6 $$\:D\left({r}_{T},{T}_{D}\right)={\sum}_{{r}_{S}}\frac{\stackrel{\sim}{A}\left({r}_{S},{T}_{D}\right)}{{m}_{tissue}\left({r}_{T}\right)}\sum\:_{i}{E}_{i}{Y}_{i}{\phi\:}_{i}({r}_{T}\:\leftarrow\:\:{r}_{S}),$$

where $\:D\left({r}_{T},{T}_{D}\right)$ is the mean absorbed dose to the target organ $\:{r}_{T}$ over the dose-integrated time interval *T*_*D*,_, *Y*_*i*_ represents the yield of radiation *i* with energy *E*_*i*_, and $\:{\phi\:}_{i}({r}_{T}\leftarrow\:\:{r}_{S})$ describes the fraction of energy absorbed by the target organ from the source tissue of radiation *i* [23]. Homogeneous activity distribution within each source organ was assumed.

For electrons, the mean emitted energy per decay *E*_*i*_, was set to 192 keV [24]. The electron self-absorbed fraction (target and source organ are the same) was assumed to be 1 for all organs besides the thyroid, for which it was calculated based on data from Endo et al. (1998) (21), using measured thyroid dimensions to estimate an effective diameter for each group. Cross-absorption of electrons between organs was assumed to be negligible.

For photons, only the most abundant photon emission (*E*_*i*_ 364 keV, *Y*_*i*_ 0.82) was considered. Photon contributions were calculated at the whole-body level, as the photon range in soft tissue (~ 10 cm) is comparable to the size of the rat and exceeds the size of individual organs. Therefore, $\:\stackrel{\sim}{A}\left({r}_{S},{T}_{D}\right)$ was derived from the whole-body activity over time using the time points 6, 24 and 144 h, with activity at *t*_*0*_ set equal to the injected activity. The photon self-absorbed fraction for the whole rat was estimated using data from Stabin and Konijnenberg (2000) (20), approximating the animals as spheres with masses corresponding to the average body weights of each group.

Estimated self-absorbed fractions for electron and photon contributions in the different study groups are displayed in Table 1. The calculations were performed in Excel and RStudio Version 4.2 [25].

### Absorbed dose estimations using Monte Carlo simulations

To complement the dose estimations described above, Monte Carlo (MC)-based dose estimations were performed. The three-dimensional particle-transport simulations were performed with the Monte Carlo N-Particle Transport (MCNP) code, version 6.3 [26] and modified ROBY rat phantoms [27, 28].

An individual ROBY phantom was generated for each study group, by scaling the organ volume parameters for the gonads, kidneys, large intestine, liver, small intestine, spleen, and thyroid based on the experimentally determined group-average organ mass. Since the ROBY phantom does not include stomach as a structure, the stomach was defined as the edge voxels of the structure stomach content, which was scaled for each study group using the average organ mass. The external body dimensions were not scaled, and the default ROBY geometry was retained. For bone marrow, brain, heart, lungs, and carcass, scaling was not possible, and the default ROBY geometry was used. Muscle, blood, and salivary glands were not available in the phantom as designated structures. Because small differences between the requested and generated organ volumes can occur during voxelization, the generated target mass was used for absorbed dose determination. Tissue specific phantom densities were applied, and the generated voxel resolution was 0.025 × 0.025 × 0.05 cm.

In total, the 12 target tissues thyroid, liver, kidney, spleen, small intestine, large intestine, gonads, stomach wall, brain, lungs, heart, and bone marrow (skeletal-marrow) were evaluated. Carcass was included in the MC simulations as a source region but not reported as an individual target region. Since muscle, blood, and salivary glands were structures not included in the phantom, they were assigned the source region carcass.

For each source region, separate MC simulations were performed for photons, internal conversion & Auger electrons, and beta particles, with 1 × 10^7^ source histories per simulation. Energies $\:{E}_{i}$ and emission yields $\:{Y}_{i}\:$were based on the ^131^I nuclear-decay data from International Commission on Radiological Protection (ICRP) Publication 107 (24). For photons and internal conversion & Auger electrons the respective energy-probability spectrum was used, while for beta particles the mean energy of 181.9 keV was used. The energy deposition in each target was scored using the MCNP *F8:p, e energy-deposition tally. Photon, internal conversion & Auger-electron, and beta components were combined using per-decay emission yields of 1.8255, 0.76208, and 1.0, respectively, to obtain yield-weighted energy deposition per nuclear transformation.

For each source-target organ pair, the MC-derived S-value, $\:S\left({r}_{T}\leftarrow\:\:{r}_{S}\right),\:$was calculated from the obtained yield-weighted energy deposited in the target region $\:{r}_{T}$ per nuclear transformation in the source region $\:{r}_{s}$, divided with the phantom-derived target organ mass $\:M\left({r}_{T}\right)$.

7 $$\:S\left({r}_{T}\leftarrow\:{r}_{s}\right)=\:\frac{1}{M\left({r}_{T}\right)}{\sum}_{i}{E}_{i}{Y}_{i}{\phi\:}_{i}({r}_{T}\:\leftarrow\:\:{r}_{S}),$$

To estimate the absorbed dose to each target region, the time-integrated activity, was determined from the experimentally derived biokinetic activity concentration data and the experimentally measured group-averaged organ mass for each source region. The absorbed dose per unit injected activity was calculated by summing the contributions from all simulated source regions, in accordance with the MIRD formalism [23]:

8 $$\:D\left({r}_{T},{T}_{D}\right)={\sum}_{{r}_{S}}\tilde A({r}_{s},\:{T}_{D})S\left({r}_{T}\leftarrow\:{r}_{s}\right),$$

ROBY phantom modifications were performed using MATLAB (MathWorks, Natick, MA, USA). MCNP result processing, dose aggregation and numerical quality assurance were performed using custom calculation scripts.

### Protein expression analysis of NIS using immunohistochemistry

To determine differences in NIS protein expression in thyroid, an additional cohort of male and female rats was intravenously injected with 0.2 mL saline at 5 or 17 weeks of age and euthanised 24 h after injection. One thyroid lobe was flash-frozen in liquid nitrogen for protein extraction and western blot analysis, while the other lobe was fixed in 4% buffered formaldehyde and embedded in paraffin.

Formalin-fixed paraffin-embedded (FFPE) thyroid tissue was sectioned into 4 μm slices and incubated for 1 h at 60 °C. One section was used for haematoxylin and eosin (H&E) staining, and another for immunohistochemistry (IHC) staining of NIS. Antigen retrieval was performed in EnVisio FLEX Target Retrieval Solution (1x Tris/EDTA, pH 9) using the DAKO PTLink (Agilent Technologies, Santa Clara, California, USA). Staining was carried out using the DAKO Autostainer Plus (Agilent Technologies), with the EnVision FLEX System (Agilent Technologies). Peroxidase blocking (Agilent Technologies) was followed by incubation with NIS primary antibody (1:300, bs-0448R, Bioss, Woburn, Massachusetts, USA) for 60 min and visualised with EnVision + HRP-labelled polymer anti-rabbit (Agilent Technologies) and DAB+ Chromogen (Agilent Technologies). Sections were counterstained with Haematoxylin (Agilent Technologies). Between each staining step, sections were washed with EnVision FLEX wash buffer (1x, Agilent Technologies).

Before imaging, sections were dehydrated in graded ethanol, followed by xylene, and cover slipped with Pertex mounting medium (Histolab Products AB). All slides were digitised using a VS200 slide scanner at 40× magnification (LRI Instruments AB, Lund, Sweden).

### Protein expression analysis of NIS using western blot

Protein was extracted from flash-frozen thyroid tissue in RIPA buffer (ThermoFisher Scientific, Waltham, Massachusetts, USA), supplemented with protease and phosphatase inhibitors (ThermoFisher Scientific) using a TissueLyser II (Qiagen, Hilden, Germany), followed by centrifugation (14,000 rpm, 10 min, 4 °C). Protein concentration was determined using the PierceTM BCA Protein Assay (ThermoFisher Scientific), and sample volumes were adjusted to load 20 µg of protein per lane. Samples were mixed 1:1 with 2× Laemmli buffer (Bio-Rad, Hercules, California, USA) and incubated at 40 °C for 20 min for denaturation. Proteins were separated on 12% Mini-PROTEAN TGX precast gels (Bio-Rad) using Tris/Glycine/SDS running buffer at 100 V for 10 min, followed by 120 V for 60 min.

Proteins were transferred to 0.2 μm nitrocellulose membranes using a Trans-Blot Turbo system (Bio-Rad) at 25 V for 7 min. Membranes were stained with Ponceau S (Thermo Fisher Scientific) to verify protein transfer and loading, followed by washing in PBS containing 0.1% Tween-20 (PBS-T).

Membranes were blocked in 5% BSA in PBS-T for 1 h at room temperature and incubated with primary antibodies for NIS (1:200, 24324-1-AP, Proteintech, Rosemont, Illinois, USA) and GAPDH (1:1000, 14C10, Cell Signaling Technology, Danvers, Massachusetts, USA) overnight at 4 °C. After washing with PBS-T, membranes were incubated with HRP-conjugated secondary antibody (1:10000, Cytiva, Marlborough, Massachusetts, USA) for 1 h at room temperature, followed by additional washing.

Proteins were detected using enhanced chemiluminescence (Clarity Western ECL substrate, Bio-Rad) and imaged using a chemiluminescence imaging system (VWR^®^ CHEMI only, VWR International, Radnor, Pennsylvania, USA). A prestained molecular weight marker (MagicMark XP, Thermo Fisher Scientific) was used to determine molecular weight. Densitometric quantification was performed using ImageJ, and the NIS signal was normalised to GAPDH from the corresponding lane after background correction.

### Statistical analysis

Data are presented as mean ± standard error of the mean (SEM). For each organ, activity concentration was analysed using a three-way analysis of variance (ANOVA) with factors time after injection, age at exposure, and sex. In addition, a two-way ANOVA with factors time and combined group (Age×Sex) was performed to assess overall differences in biokinetic profiles between groups. When a significant main or interaction effect was observed, post hoc multiple comparisons were performed using Tukey’s range test.

All analyses were performed using GraphPad Prism (Version 10.3.1, GraphPad Software, Boston, MA, USA). The significance level was set at *p* < 0.05.

## Results

### Biodistribution of ^131^I is influenced by age at exposure and sex

**Fig. 1:**
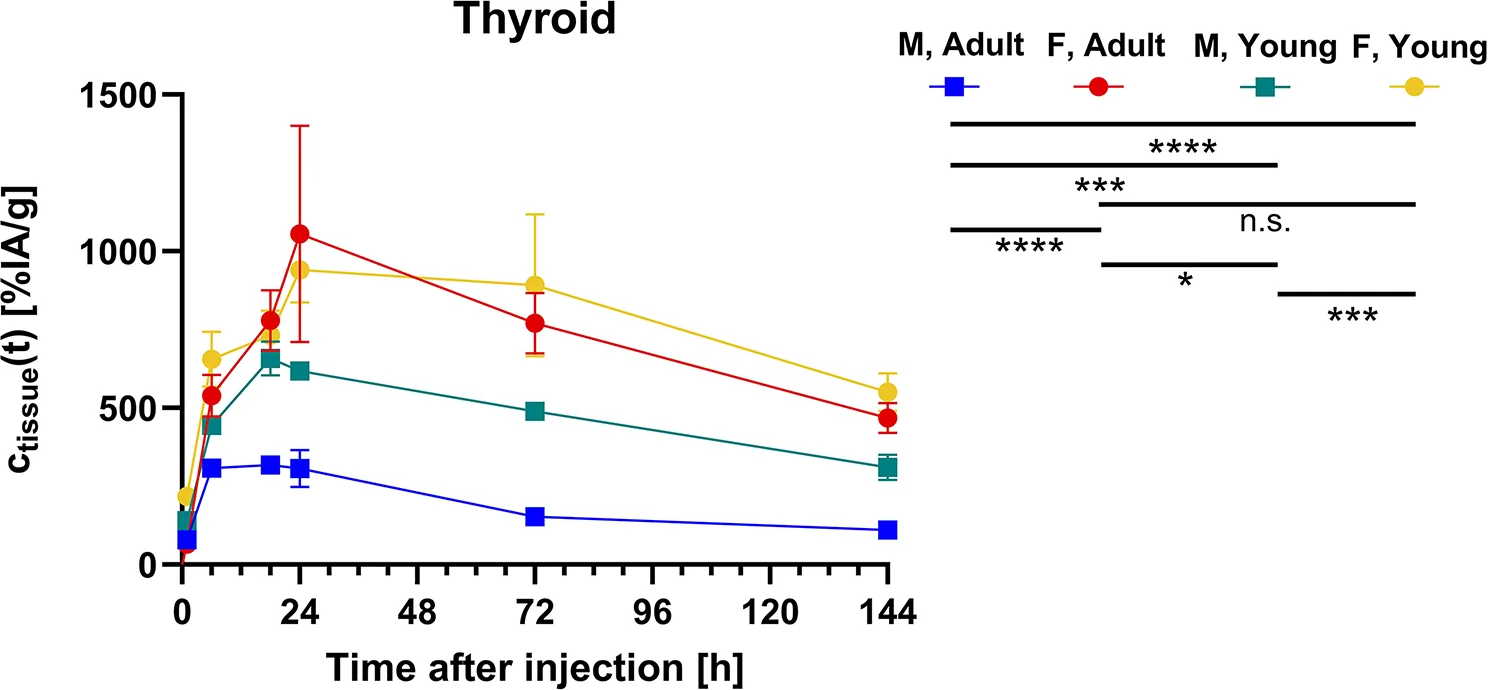
^131^I activity concentration c_tissue_(t) in %IA/g over time in thyroid. The data is corrected for radioactive decay to the time of injection, in order to demonstrate the biodistribution of iodine. The different study groups are represented with different colours. Statistical significance between the different biokinetic profiles of the study group was assessed using two-way ANOVA, followed by Tukey’s post hoc test at *p* < 0.05. Data is presented as the mean ± SEM. **** *p* < 0.0001, *** *p* < 0.001, ** *p* < 0.01, * *p* < 0.05, n.s. *p* > 0.05

**Fig. 2:**
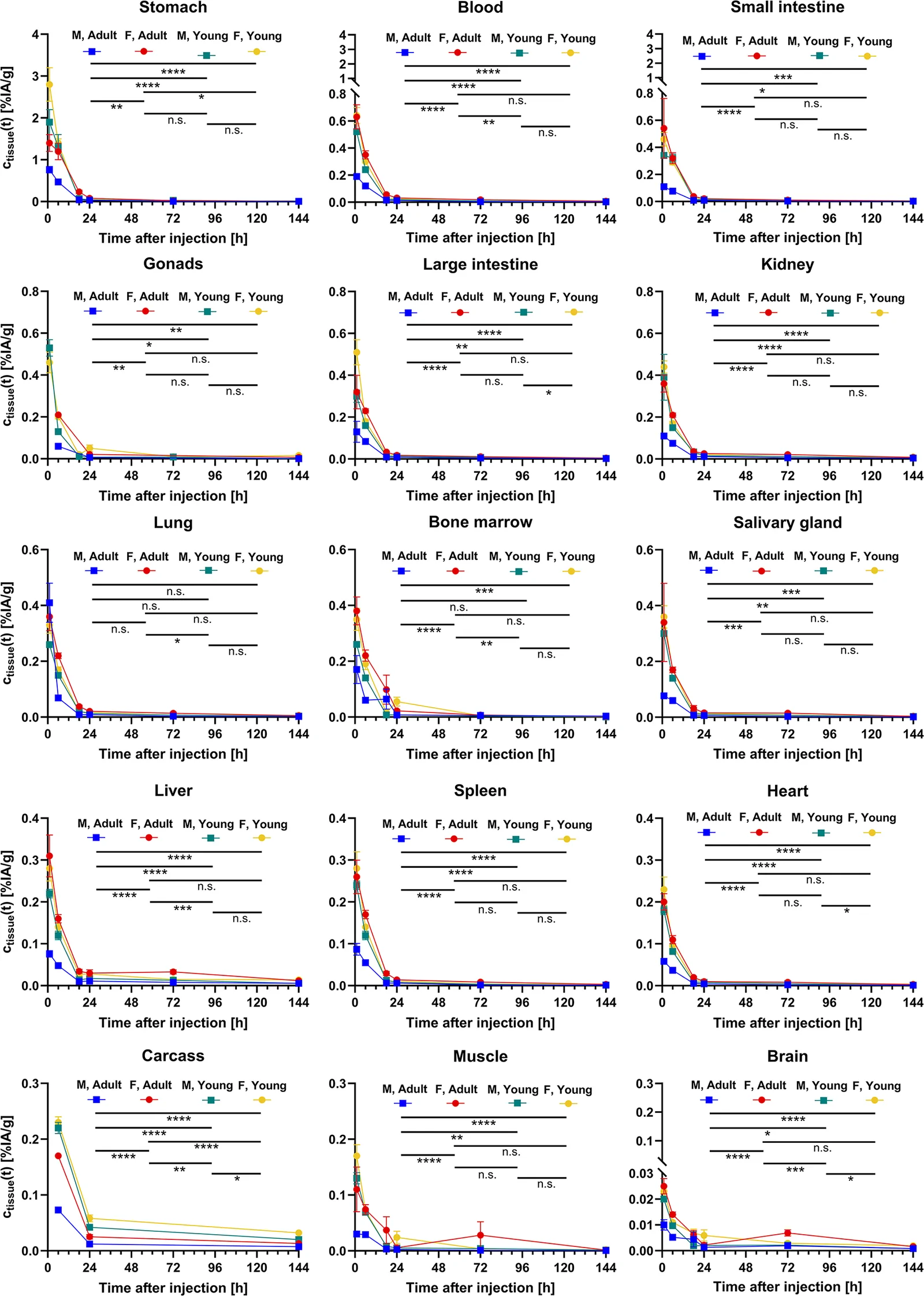
^131^I activity concentration c_tissue_(t) in %IA/g over time for non-thyroid tissues. The data is corrected for radioactive decay to the time of injection, in order to demonstrate the biodistribution of iodine. For each tissue, the different study groups, represented by different colours, are shown in a single graph. Statistical significance between the different biokinetic profiles of the study group was assessed using two-way ANOVA, followed by Tukey’s post hoc test at *p* < 0.05. Data is presented as the mean ± SEM. **** *p* < 0.0001, *** *p* < 0.001, ** *p* < 0.01, * *p* < 0.05, n.s. *p* > 0.05

The biodistribution data, presented as the ^131^I activity concentration per injected activity (%IA/g) over time, decay corrected to time of injection, are shown in Fig. 1 for the thyroid and Fig. 2 for all non-thyroid tissues. Exact numerical values and results from statistical tests are available in Additional files 1 and 2.

In all groups, the thyroid exhibited the highest activity concentration. In males, the maximum activity was measured at 18 h, whereas in females, it was observed at 24 h, independent of age at exposure. Female rats showed a longer uptake phase of ^131^I into the thyroid, followed by prolonged retention. All other tissues exhibited substantially lower activity concentrations, up to 3 orders of magnitude lower than the thyroid at peak levels. Maximum activity in non-thyroid tissues was observed at 1 h post-injection, followed by a continuous decline. Among these tissues, the stomach showed the highest activity concentrations, while the brain had the lowest.

Three-way ANOVA demonstrated a significant main effect of time in all tissues (*p* < 0.0001), with large effect sizes (η² = 0.325–0.826). A significant main effect of sex was also observed across all tissues (*p* < 0.0148), with smaller effect sizes (η² = 0.006–0.204), and the largest effect in the thyroid. In contrast, age showed a significant main effect only in a subset of tissues and with the smallest effect sizes (η² = 0.005–0.101). Significant Time×Sex interactions were observed across all tissues, whereas other interactions were present only in selected tissues. Post hoc comparisons identified multiple significant pairwise differences within time points, most frequently at early time points (1–6 h), primarily involving male adult rats compared to the other groups. For the thyroid, gonads, and carcass, significant differences were also observed at later time points (≥ 24 h).

Two-way ANOVA, combining sex and age into a single group factor, confirmed the strong effect of time across all tissues (all *p* < 0.0001, η² = 0.59–0.83). A significant main effect of group was observed in all tissues (*p* ≤ 0.0394), although with smaller effect sizes (η² = 0.008–0.2798). Significant Time×Group interactions were detected for all tissues (*p* ≤ 0.0001). Group-level post hoc analysis, averaging across time points, showed significant differences between at least two groups in all tissues (Figs. 1 and 2). This analysis was used to assess overall differences between sex and age groups independent of time-dependent variation. Male adult rats generally exhibited lower activity concentrations than female adult, male young, and/or female young rats across most tissues (all *p* < 0.01). Differences between male young and female young rats were less consistent and were only observed in a limited number of tissues, including the brain, carcass, heart, large intestine, and thyroid. The largest group differences were observed in the thyroid, where female rats showed substantially higher activity concentrations than male adult rats, and male young rats also differed from female young rats.

### Absorbed doses of ^131^I to tissues are influenced by age at exposure and sex

**Fig. 3:**
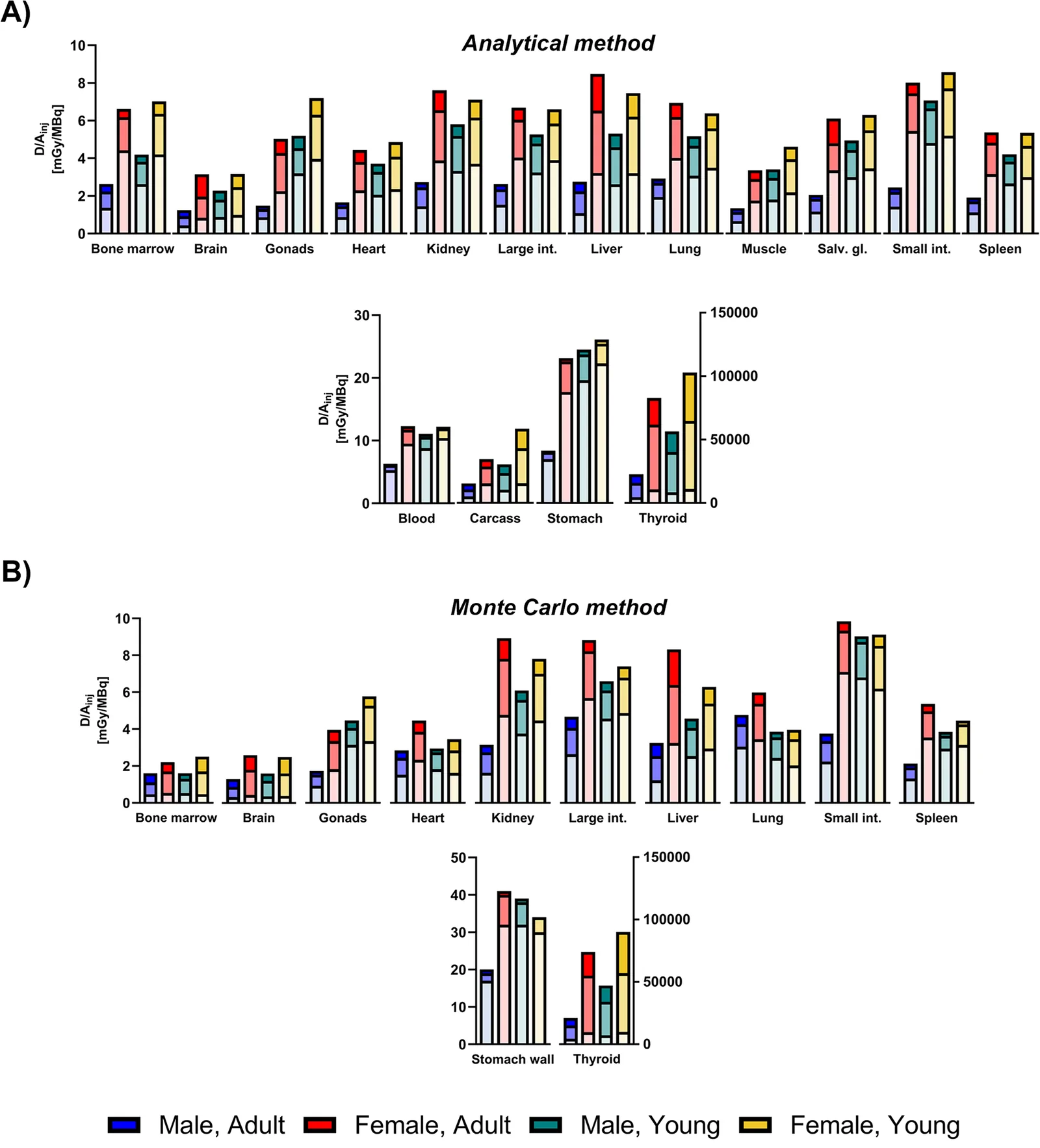
Mean absorbed dose per unit injected ^131^I activity, D/Ainj, for the different tissues under investigation using two dosimetric approaches. **A**) D/A_inj_ estimated using the analytical method. **B**) D/A_inj_ estimated using Monte Carlo simulations. For each tissue, the different study groups are presented in different colours. The bottom segment of each bar represents the value after 24 h, while the bottom and middle segments combined indicate the absorbed dose per injected activity after 144 h. The total bar presents the absorbed dose per injected activity towards infinity

The estimated mean absorbed dose per unit injected ^131^I activity, D/A_inj_ [mGy/MBq], to the various tissues over time is displayed in Fig. 3 for the different groups. Exact numerical values are available in Additional file 3 for the analytical method and Additional file 4 for the MC method. The fitted biokinetic curves used for absorbed dose estimation, together with the measured activity data decay corrected to the time of euthanasia, are shown in Additional file 5 and the corresponding model parameters are provided in Additional file 6.

The thyroid received the highest D/A_inj_ in all study groups at all time points, irrespective of the dosimetric method. Towards infinity, female rats exposed at a young age exhibited the highest absorbed dose to the thyroid of 100 Gy/MBq and 90 Gy/MBq, using the analytical and MC-based approaches, respectively. This was followed by adult females (83 Gy/MBq and 74 Gy/MBq), young males (56 Gy/MBq and 47 Gy/MBq), and adult males (23 Gy/MBq and 21 Gy/MBq), demonstrating a pronounced difference in the absorbed dose to the thyroid between the sexes in both age groups, irrespective of dosimetric method.

Among non-thyroid tissues, the highest D/A_inj_ was observed in the stomach for all groups, regardless of dosimetric approach. Using the analytical method, the brain received the lowest absorbed dose in all study groups. With the MC method, the lowest absorbed dose was observed in the brain for adult males, in the bone marrow for adult females, and in the brain and bone marrow for both young groups. Other organs with relatively high D/A_inj_ values using the analytical method included the lung, liver and kidney for male adult rats, small intestine, kidney and liver for male young rats, liver, small intestine and kidney for female adult rats, and small intestine, ovaries, and liver for female young rats. For the MC-based approach, however, other organs with relatively high D/A_inj_ values included the lung, small and large intestines for male adult rats, small intestines, large intestines, and kidney for male young rats, as well as small intestine, kidney, and large intestines for both female groups. In general, D/A_inj_ across all non-thyroid tissues was several orders of magnitude lower than that of the thyroid, regardless of the dosimetric approach.

Male adult rats received the lowest D/A_inj_, whereas female young or adult rats displayed the highest D/A_inj_ in most non-thyroid tissues. The difference in D/A_inj_ between the two female cohorts, estimated using the analytical method, was on average 1.4 mGy/MBq, while the corresponding difference between the male cohorts was 1.7 mGy/MBq.

### NIS expression in thyroid tissue

**Fig. 4:**
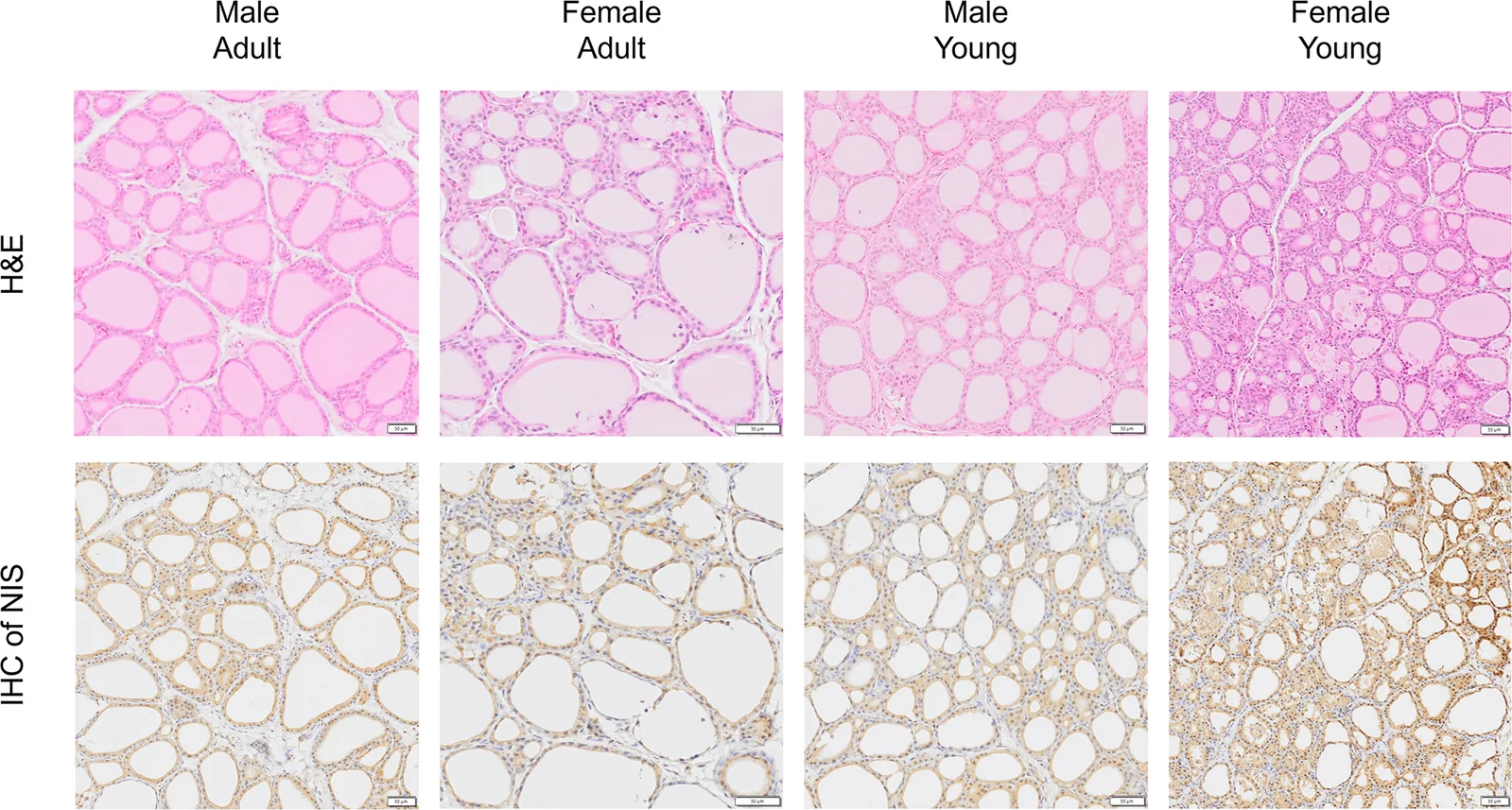
Representative H&E and NIS immunohistochemical staining of rat thyroid tissue. Representative H&E (top) and NIS IHC (bottom) staining of thyroid tissue from adult and young, male, and female rats. H&E confirmed normal follicular structure across all groups. NIS immunoreactivity is observed in follicular cells. Scale bars: 50 μm

Representative H&E and NIS IHC stainings of thyroid tissue are shown in Fig. 4 for the different age and sex groups. H&E stainings confirmed normal follicular architecture across all groups. NIS immunoreactivity was clearly observed in thyroid follicular cells in all groups, showing predominantly cytoplasmic staining. Staining intensity ranged from low to moderate and varied considerably between samples. Changes in the staining protocol, antibody provider and antibody concentration did not improve signal intensity or reduce variability.

Due to low signal intensity and high variability between samples, reliable quantitative assessment of NIS expression by IHC was not feasible. Therefore, IHC was used solely for qualitative assessment of NIS localisation, and additional quantification was performed using western blotting.

**Fig. 5:**
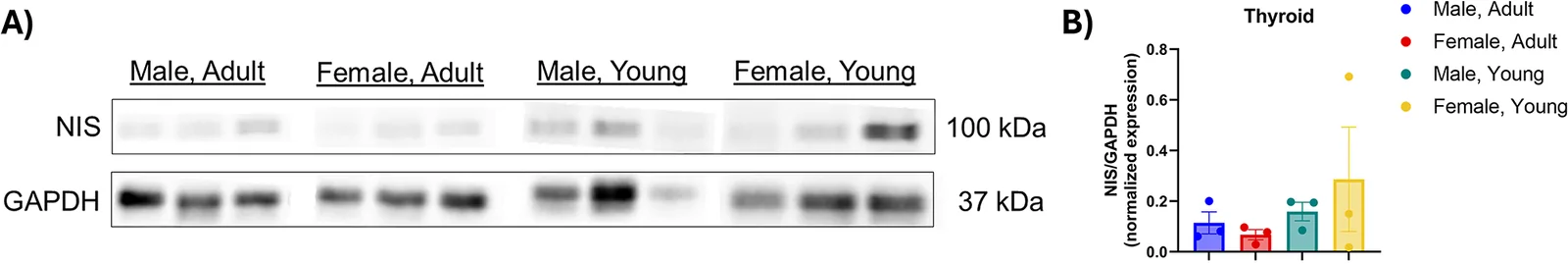
NIS protein expression in thyroid tissue assessed by western blot. **A**) Representative western blot images of NIS protein expression in thyroid tissue from male adult, female adult, male young, and female young rats. GAPDH (37 kDa) was used as a loading control. **B**) Densitometric analysis was performed, and relative NIS expression was quantified based on the mature, fully glycosylated NIS band (100 kDa) and normalised to GAPDH. Data are presented as mean ± SEM

Western blot analysis of thyroid protein extracts (Fig. 5, full membranes in Additional file 7) confirmed the presence of NIS in all groups. Densitometric quantification of band intensity indicated variability between groups, with a tendency toward higher expression in young animals than in adults. However, variability between individual samples was observed, and quantitative analysis revealed no consistent statistically significant differences. Overall, the western blot results supported the qualitative IHC findings, indicating the presence of NIS in all groups, with no clear, consistent differences in expression levels.

## Discussion

When studying the radiobiological effects of ^131^I on the thyroid, age and sex are two important factors that should be taken into consideration, as demonstrated after the Chernobyl accident, where an increase in thyroid cancer was observed specifically among young girls [8–10]. The results of the present study support a sex-dependent difference in response to ¹³¹I in rats, demonstrating that female rats exhibit higher uptake and prolonged retention of ¹³¹I in the thyroid, resulting in a substantially higher absorbed dose than males. An age difference, on the other hand, was primarily evident in male rats.

Despite increasing awareness of sex differences in biology and physiology, a notable sex bias still exists in preclinical research, where male rodents are frequently used as the default model and sex-specific analyses are often lacking [29]. Although the Chernobyl accident clearly showed an increase in thyroid cancer in young girls, many experimental studies have still been performed in male rodents e.g. [30, 31]. The present findings show that using male animals as the default model may significantly underestimate thyroid uptake and absorbed dose when extrapolating data to females.

As expected, the thyroid exhibited the highest activity concentration across all time points and exposure groups and was the only organ with a distinct uptake phase. Female rats, independent of age, showed a longer uptake phase lasting up to 24 h after injection, whereas males showed increased uptake only up to 18 h. These findings are consistent with previous biodistribution studies in male and female rats of different ages [11, 13]. The differences in uptake and retention of ^131^I in the thyroid between the sexes resulted in a ~ 3.6-fold higher absorbed dose per injected activity to the thyroid in adult female rats and a ~ 1.8-fold higher value in young female rats compared with males of the same age.

The observed differences in thyroid uptake between the sexes cannot be explained solely by differences in body weight and thyroid size, although these factors may contribute. At the time of exposure, male adult rats had higher body weight than females but exhibited lower activity concentrations, whereas male young rats also showed lower uptake than female adults, despite having lower body weight. This suggests that the differences between male and female rats are primarily biological and sex-related, rather than due to weight differences. Likewise, the two female study groups were closer in weight than the male groups, and the difference in average activity concentration between the females was not statistically significant. This could indicate that the difference in thyroid activity concentration between the two male groups was more likely due to differences in weight and, potentially, thyroid size.

Previous studies have also confirmed significant sex-specific differences in body weight and thyroid size in rats, both of which increase with age. However, relative thyroid weight was not significantly affected by age in either sex [32]. In addition, the structure of the rat thyroid appears to differ between males and females, with females having a significantly higher relative thyroid weight with smaller follicular cells of lower volume compared to males, although the overall ratio of follicular cells within the thyroid is similar [33].

Rather than size alone, sex-related differences in thyroid uptake may be explained by hormonal regulation and thyroid physiology, as the influence of gonadal hormones on thyroid function has been widely reported. Both testosterone and estradiol appear to regulate thyroid growth in rats by influencing thyroid-stimulating hormone (TSH) secretion and TSH receptor regulation [34, 35]. Estradiol in particular has been suggested to stimulate thyroid growth, in a sex-specific manner. Thyroid function also decreases with age in rats, and these age-related changes appear to be influenced by sex, suggesting that gonadal hormones impact thyroid function [36]. The effects of estrogen on thyroid activity and radioiodine uptake have been extensively studied due to the higher incidence of thyroid diseases in women, but contradictory results have been reported depending on age and gonadal status [37, 38]. However, several studies have found that the ^131^I uptake in female rats is highest during the estrous phase, and lowest during the diestrus phase [38–40]. In addition to hormonal influences, differences in metabolic rate between the sexes can also affect the uptake of ^131^I. In general, metabolic rates decrease with the age of the animal, both in males and females, and tend to be lower in males compared to females [41–43].

Since iodine uptake is mediated by NIS, differences in NIS expression or activity could also contribute to the observed variation. In the present study, NIS was detected in the thyroid of all groups by both IHC and western blot analysis; however, no consistent differences in NIS protein expression were observed. This suggests that differences in thyroidal iodine uptake may not be explained by total NIS abundance. Iodine uptake into the thyroid is not only dependent on protein expression of NIS, but also on the electrochemical sodium gradient generated by the Na^+^/K^+^-ATPase [3]. In addition, only basolateral membrane localisation of NIS contributes to active iodide transport.

However, based on the present staining approach, differences in membrane localisation between study groups could not be clearly distinguished. Since NIS is known to be distributed both intracellularly and at the plasma membrane, the observed variability in staining patterns between samples may reflect differences in cellular localisation or regulation of NIS trafficking rather than differences in total protein expression alone. In addition, immature or partly glycosylated forms of NIS have previously been associated with intracellular localisation rather than functionally active membrane-bound protein [44, 45]. The relatively high variability in NIS expression between samples also indicates that additional replicates may be necessary to draw more definitive conclusions. Altogether, these findings suggest that the observed differences in thyroidal iodine uptake are not solely explained by differences in total NIS expression but may instead be related to differences in basal membrane NIS expression or functional regulation of iodide transport or other physiological factors.

Among non-thyroid tissues, the stomach exhibited the second-highest activity concentration and absorbed dose per injected activity in all exposure groups independent of dosimetric method, which was expected due to the high expression of NIS in this organ [46]. The small and large intestines also showed comparably higher activity concentrations and absorbed doses, consistent with reported NIS expression in these tissues [46]. In contrast, the low activity concentration and absorbed dose in the brain are plausible, since ¹³¹I cannot pass the blood–brain barrier [47]. Based on the literature, salivary glands also express NIS to a high degree [46] and would therefore be expected to show higher activity concentrations and absorbed doses than observed in the present study. One possible explanation is that only the submandibular glands were collected, whereas previous studies have demonstrated higher accumulation of radioiodine and more pronounced dysfunction in the parotid glands compared with the submandibular glands [48]. Interestingly, that study also showed that the cumulative ^131^I radioactivity in the submandibular glands increased with patient age.

For both young male and female rats, the sex organs were among the tissues with the highest activity concentrations. Overall, ovaries exhibited higher activity concentrations and absorbed doses than testes at most investigated time points, although testes showed slightly higher activity concentrations 1 h after injection compared to ovaries in the young groups. In adult rats, absorbed doses to the gonads were lower than in the young groups, which may partly be explained by the lack of a 1 h time point for dose estimation and, therefore a potential underestimation of the absorbed dose. Both ovaries and testes express NIS [46], and a previous study in female rats found that the NIS activity in the ovaries was influenced by the estrogen cycle, with NIS upregulated immediately before ovulation when estrogen levels are high [49]. Since the female rats in the present study may have been in different phases of their estrogen cycle, this could have contributed to variability in ^131^I uptake at the individual level.

Several methodological factors should be considered when interpreting the results. The number and timing of sampling points influence the accuracy of the time–activity curves. In the present study, six sampling time points were included, and the fitted biexponential curves generally provided a good description of the measured activity data across the investigated tissues. However, the thyroid was the only organ exhibiting a distinct uptake phase followed by a slower clearance phase, making the timing of sampling particularly important. Consequently, only three and four data points were available to characterise the post-peak clearance phase in female and male thyroids, respectively. Although these measurements permitted fitting of the biokinetic curves and estimation of the time-integrated activity concentration in the thyroid, the limited number of post-peak measurements may have reduced the robustness of the model and introduced additional uncertainty into the estimated absorbed dose to the thyroid. Additional time points between 24 and 72 h or at later stages would therefore improve the accuracy of the thyroid dose calculation. Dietary iodine intake is another important factor affecting thyroid uptake of ¹³¹I. Different amounts of stable iodine impact iodine saturation in the thyroid and further iodine uptake [17]. Previous studies have demonstrated that iodine-deficient diets (0.05–0.1 mg/kg stable iodine) can substantially increase thyroid uptake but also inter-individual variability [13, 14]. Since food intake was not monitored in the present study, differences in stable iodine intake between individuals may have contributed to the observed variability between individuals.

Finally, although the contribution of photons emitted by ^131^I is often considered negligible in small-animal dosimetry, it may contribute substantially to the absorbed dose in extrathyroidal tissues with low iodine uptake and retention. The MC simulations performed in this study demonstrated that, on average across all tissues, photons contribute to 24–31% of the absorbed dose, but only about 1% in the thyroid and 2–4% in the stomach. In contrast, the dose contributions from photons were highest in tissues with low local ^131^I uptake, accounting for 65–77% of the absorbed dose to bone marrow and 78–91% of the absorbed dose to the brain. Similarly, the importance of cross-irradiation depends strongly on ^131^I tissue uptake. The absorbed dose to the thyroid (99.9% self-dose, 0.1% cross-dose) and stomach (92–95% self-dose, 5–8% cross-dose) were predominantly caused by self-irradiated, whereas bone marrow (1% self-dose, 99% cross-dose) and brain (9–23% self-dose, 77–91% cross-dose) received most of their absorbed dose through cross-irradiation. The carcass was the largest source of cross-dose for most tissues. These results confirm that local energy deposition dominates in tissues with high ^131^I uptake, like the thyroid and stomach, whereas cross-irradiation becomes increasingly important in tissues with low uptake and retention, like brain and bone marrow. Regardless, caution is required when translating the results to the human system, as the contribution of photons will be higher in humans [50].

Despite differences in radiation-transport modelling, the MC-derived dose estimates showed a broadly similar overall pattern to those obtained using the analytical method, and the relative differences between study groups were generally preserved. Differences in absorbed dose estimates may partly reflect differences in the treatment of radiation transport and absorbed fractions. The analytical method provides a straightforward approach but simplifies radiation transport by assuming complete electron self-absorption for all tissues except the thyroid, neglecting electron cross-dose and approximating photon absorption at the whole-body level. In contrast, the MC method explicitly accounts for source-to-target energy transport and cross-irradiation but depends on the anatomical geometry of the ROBY phantom. Although several organs were scaled according to experimentally determined group-average organ masses, the external body dimensions and several organs were not scaled and retained the default ROBY geometry. Furthermore, blood, muscle, and salivary glands were not represented as dedicated structures and their activity was assigned to the carcass, while the stomach geometry was defined based on the stomach content. These limitations may introduce uncertainty in source-to-target distances and energy deposition and may partly explain differences in results from the two dosimetric approaches. Nevertheless, both approaches supported the pronounced sex- and age-dependent differences across tissues.

An important consideration of the biokinetic results presented in this study is that the activity concentrations were corrected for the physical decay of ^131^I and therefore primarily reflect the biological uptake, distribution and retention of iodine. Since radioiodine isotopes share similar chemical properties, the biodistribution patterns described in this study are also relevant and easily transferable to other radioiodine isotopes used in nuclear medicine and research, such as ^123^I, ^124^I, and ^125^I by adding physical decay. However, the resulting absorbed doses for these iodine isotopes cannot be calculated using only the data presented here and their physical decay data; instead, additional calculations are required for this purpose including the physical emission data.

When comparing the results with previous rodent studies, similar overall trends in ¹³¹I biodistribution were observed. Previous studies in both male and female rats reported peak thyroid activity concentrations between approximately 12 and 24 h after injection, and overall similar distribution patterns between organs [11, 13, 14]. However, differences in absolute activity concentrations and absorbed doses between studies were observed. Hamilton et al. (1953), who investigated adult female Long-Evans rats, reported overall higher thyroid activity concentrations than observed in the present study and a decrease from 9 to 13 h post-injection. Activity concentrations in other tissues showed a similar decrease starting from the 1-h time point. Lee et al. (1979) reported maximum thyroid activity concentrations between 12 and 17 h after injection in female 6-week-old Long-Evans rats. In addition, higher administered activity resulted in a longer effective half-life and uptake phase, although without significant changes in absorbed dose per injected activity. In contrast, our previous study in young male rats showed considerably lower thyroid uptake and absorbed doses compared with the present findings, and an additional iodine uptake phase in the stomach, small intestines, spleen, and muscle [11]. Studies in mice generally reported higher activity concentrations and absorbed doses than studies in rats [12, 15, 16], which might be explained by differences in body size and metabolic rate [51].

One likely explanation for these differences between studies is variation in dietary iodine intake, as discussed before. In addition, circadian variation has been shown to influence iodine uptake in rodents [12, 14, 18, 19], with increased thyroid activity during their active phase, demonstrating differences in uptake depending on the time of administration. Since injections in the present study were performed during the day over an 8-h period, the influence of circadian variation cannot be excluded. Altogether, comparison of the different studies demonstrates that factors such as age, sex, activity administered, species, diet and circadian rhythm significantly impact the biodistribution of ^131^I. This highlights the importance of detailed reporting of experimental parameters, including exact age, body weight, and dietary conditions, to improve reproducibility and comparability between studies.

Thyroid dosimetry studies in ^131^I fallout-affected regions following the Chernobyl accident have additionally demonstrated pronounced age- and sex-dependent differences when it comes to the absorbed dose to the thyroid. Generally, high variability was found between exposed individuals, but the absorbed dose to the thyroid in young children was several-fold higher than in adults. Sex-specific differences were primarily observed during adolescence, with males having higher ^131^I activity compared to females [52]. More recent large-scale dosimetry studies further emphasised the impact of thyroid mass, dietary iodine intake, and individual dietary behaviour on the absorbed dose as well as their uncertainties [53]. Interestingly, a higher absorbed dose to thyroid was estimated in boys compared with girls in the investigated exposed populations. However, these differences were primarily attributed to differences in dietary behaviour and milk consumption rather than intrinsic biological differences.

Another difference between the studies conducted in the aftermath of the Chernobyl accident and the biodistribution study presented here lies in the route of exposure. In contrast to intravenous administration, the uptake of ^131^I from the Chernobyl accident occurred primarily through the consumption of contaminated food, especially milk, and with a small contribution from inhalation. Once absorbed in the gastrointestinal tract, however, ^131^I rapidly enters the systemic circulation, from which its subsequent distribution follows patterns similar to those described here. Although intravenous administration bypasses the gastrointestinal absorption phase, this study thus provides information on the systemic biodistribution of ^131^I after it enters the bloodstream.

### Perspectives and significance

The results of the study highlighted the importance of considering sex and age at exposure for both experimental small-animal dosimetry and potentially clinical dosimetry. Current dosimetry approaches often rely on simplified or sex-independent guidelines, despite clear evidence that ¹³¹I uptake and retention differ substantially between biological groups. Especially in the context of radiological protection following a nuclear accident or emergency, age and sex represent two easily assessable factors that could improve risk assessment and guide appropriate follow-up and care. The strong impact of sex, especially in thyroid uptake and retention, might be particularly relevant for understanding differences in radiation response and susceptibility to thyroid diseases following ¹³¹I exposure. In addition, the lack of consistent differences in total NIS expression suggests that functional regulation of iodide transport, rather than protein abundance alone, may play an important role in thyroid iodine kinetics. Future studies examining hormonal regulation, metabolism, and long-term biological responses at the genomic level following ¹³¹I exposure could help clarifying the mechanisms underlying these observed differences. Such studies may contribute to improved individualised radionuclide therapy and more accurate risk assessment following nuclear incidents.

## Conclusions

In conclusion, the present study demonstrates that the biodistribution and dosimetry of ¹³¹I are influenced by both sex and age at exposure, with the most pronounced differences observed in the thyroid. Female rats exhibited higher and prolonged thyroid uptake of ¹³¹I, resulting in increased absorbed doses compared with males, whereas age-related differences were primarily evident in male rats. These findings demonstrate that sex- and age-dependent variation in iodine biokinetics should be considered in thyroid dosimetry, both in the context of radionuclide therapy and radiation risk assessment following nuclear emergencies.

## Supplementary Information

Below is the link to the electronic supplementary material.

Additional file 1: ¹³¹I activity concentrations in tissues over time for the different study groups. Description: Table of mean decay-corrected ¹³¹I activity concentrations (%IA/g) measured in organs and tissues of male and female Sprague–Dawley rats exposed at 5 weeks of age (Table 1) and 17 weeks of age (Table 2).

Additional file 2: Statistical analyses of ¹³¹I biodistribution data. Description: Full statistical outputs of 2-way and 3-way ANOVA analyses for all investigated tissues and study groups, including Tukey’s multiple comparison tests for statistically significant comparisons.

Additional file 3: Mean absorbed dose per unit injected activity in tissues of the different study groups, determined by the analytical method. Description: Table of mean absorbed dose per unit injected activity (D/A_inj_) in organs and tissues of male and female Sprague–Dawley rats exposed to ¹³¹I at 5 weeks of age (Table 1) and 17 weeks of age (Table 2), determined by the analytical method.

Additional file 4: Mean absorbed dose per unit injected activity in tissues of the different study groups, determined by the MC-method. Description: Table of mean absorbed dose per unit injected activity (D/A_inj_) in organs and tissues of male and female Sprague–Dawley rats exposed to ¹³¹I at 5 weeks of age (Table 1) and 17 weeks of age (Table 2), determined by the MC method.

Additional file 5: Biokinetic curves fitted to ^131^I activity concentrations in examined tissues. Description: Figures showing measured ^131^I activity concentrations together with the fitted biexponential biokinetic curves for examined tissues of male and female Sprague–Dawley rats exposed at 5 or 17 weeks of age. The fitted equations used for time-integrated activity concentration estimation are shown for each study group. The file also includes the fitted whole-body activity curves used to estimate the photon contribution to the absorbed dose.

Additional file 6: Biokinetic curve parameters for ^131^I activity concentrations. Description: Fitted biexponential curve parameters (A, B, λ₁, and λ₂) for examined tissues of male and female Sprague–Dawley rats exposed at 5 or 17 weeks of age. Separate worksheets are provided for each study group and include the whole-body biokinetic curves used to estimate the photon contribution to the absorbed dose.

Additional file 7: Full western blot membrane images for NIS protein expression analysis. Description: Uncropped western blot membrane images showing Ponceau S staining, GAPDH loading controls, and NIS protein expression in thyroid tissue samples from male and female Sprague–Dawley rats at 5 or 17 weeks of age.

## Data Availability

The raw datasets supporting the findings of this study are available from the corresponding author upon request.
