# Supplementary material for: Sex and age at exposure influence 131I biodistribution and dosimetry in Sprague–Dawley rats
Source: Biol Sex Differ. 2026 Sep 18;17:154. doi: 10.1186/s13293-026-00989-4 (PMC13589867; doi:10.1186/s13293-026-00989-4)
Supplement: Supplementary file 1 [file 13293_2026_989_MOESM1_ESM.pdf]

Additional file 1

^{131}I activity concentrations in examined tissues over time in young and adult male and female rats.

Mean decay-corrected ^{131}I activity concentrations, $c_{tissue}(t)$, expressed as percentage of injected activity per gram tissue (%IA/g), measured in examined organs and tissues of male and female Sprague–Dawley rats exposed to ^{131}I at 5 (young) or 17 (adult) weeks of age. Measurements were obtained at six time points ranging from 1 h to 144 h post-injection. Data are presented as mean (SEM).

For the adult exposure groups, rats in the 1-hour group were not euthanised exactly 1 h after injection. "nd" indicates that no samples were collected at the corresponding time point.

Table S1: ^{131}I activity concentration, c_{tissue}, over time in male and female rats exposed at an age of 5 (young) weeks. The activity concentration is decay corrected to time of injection and displayed for 6 measured time points ranging from 1 hour to 144 hours for examined organs and tissues. The injected activity was on average 0.36 (SEM = 0.05) MBq.

	$c_{tissue}(t)$ [%IA/g]					
Tissue	1 hour	6 hours	18 hours	24 hours	72 hours	144 hours
Male, Young						
Blood	0.52 (0.02)	0.24 (0.01)	0.020 (0.001)	0.019 (0.002)	0.010 (0.001)	0.0042 (0.0006)
Bone marrow	0.26 (0.01)	0.14 (0.01)	0.0078 (0.0013)	0.0077 (0.0008)	0.0043 (0.0004)	0.00011 (0.00002)
Brain	0.020 (0.001)	0.0097 (0.0009)	0.0019 (0.0003)	0.0020 (0.0002)	0.0022 (0.0002)	0.00083 (0.00007)
Carcass	nd	0.22 (0.01)	nd	0.042 (0.005)	nd	0.020 (0.001)
Gonads	0.53 (0.04)	0.13 (0.01)	0.011 (0.001)	0.0076 (0.0006)	0.0084 (0.0017)	0.0015 (0.0002)
Heart	0.18 (0.01)	0.082 (0.006)	0.0086 (0.0003)	0.0078 (0.0007)	0.0050 (0.0004)	0.0011 (0.0000)
Kidney	0.39 (0.11)	0.15 (0.01)	0.033 (0.015)	0.016 (0.001)	0.012 (0.002)	0.0046 (0.0006)
Large intestine	0.30 (0.03)	0.16 (0.01)	0.014 (0.001)	0.013 (0.002)	0.0075 (0.0019)	0.0026 (0.0003)
Liver	0.22 (0.01)	0.12 (0.01)	0.014 (0.001)	0.017 (0.002)	0.013 (0.001)	0.0055 (0.0006)
Lung	0.26 (0.01)	0.15 (0.01)	0.021 (0.007)	0.013 (0.001)	0.0073 (0.0005)	0.0038 (0.0004)
Muscle	0.13 (0.01)	0.069 (0.004)	0.0083 (0.0012)	0.0052 (0.0005)	0.0042 (0.0004)	0.0013 (0.0001)
Salivary gland	0.30 (0.01)	0.14 (0.01)	0.013 (0.001)	0.011 (0.001)	0.0067 (0.0007)	0.0030 (0.0006)
Small intestine	0.34 (0.01)	0.32 (0.03)	0.016 (0.001)	0.015 (0.001)	0.0065 (0.0014)	0.0022 (0.0003)
Spleen	0.24 (0.01)	0.12 (0.01)	0.013 (0.003)	0.0078 (0.0008)	0.0032 (0.0003)	0.0015 (0.0002)
Stomach	1.9 (0.3)	1.3 (0.3)	0.073 (0.004)	0.039 (0.004)	0.027 (0.018)	0.0038 (0.0006)
Thyroid	140 (25)	440 (20)	660 (54)	620 (18)	490 (20)	310 (40)
Female, Young						
Blood	0.64 (0.06)	0.30 (0.01)	0.030 (0.003)	0.025 (0.004)	0.0089 (0.0005)	0.0081 (0.0012)
Bone marrow	0.35 (0.04)	0.19 (0.02)	0.015 (0.003)	0.055 (0.016)	0.0045 (0.0003)	0.00045 (0.00013)
Brain	0.023 (0.002)	0.011 (0.001)	0.0068 (0.0016)	0.0059 (0.0022)	0.0029 (0.0005)	0.0019 (0.0003)
Carcass	nd	0.23 (0.01)	nd	0.058 (0.006)	nd	0.032 (0.001)
Gonads	0.46 (0.05)	0.20 (0.01)	0.020 (0.003)	0.05 (0.016)	0.010 (0.002)	0.016 (0.006)
Heart	0.23 (0.03)	0.096 (0.004)	0.013 (0.001)	0.010 (0.001)	0.0047 (0.0003)	0.0038 (0.0006)
Kidney	0.44 (0.03)	0.17 (0.01)	0.024 (0.003)	0.022 (0.004)	0.011 (0.001)	0.0096 (0.0014)
Large intestine	0.51 (0.06)	0.18 (0.01)	0.021 (0.007)	0.017 (0.002)	0.0062 (0.0003)	0.0052 (0.0005)
Liver	0.28 (0.03)	0.14 (0.01)	0.027 (0.004)	0.028 (0.004)	0.015 (0.001)	0.014 (0.003)
Lung	0.3 (0.03)	0.17 (0.01)	0.019 (0.002)	0.017 (0.002)	0.0070 (0.0001)	0.0064 (0.0008)
Muscle	0.17 (0.02)	0.072 (0.003)	0.0076 (0.0011)	0.024 (0.011)	0.0037 (0.0011)	0.0022 (0.0003)
Salivary gland	0.36 (0.04)	0.17 (0.01)	0.015 (0.001)	0.015 (0.002)	0.0079 (0.0016)	0.0045 (0.0007)
Small intestine	0.46 (0.05)	0.29 (0.02)	0.027 (0.002)	0.021 (0.002)	0.011 (0.005)	0.0060 (0.0010)
Spleen	0.28 (0.04)	0.14 (0.01)	0.011 (0.001)	0.011 (0.002)	0.0037 (0.0006)	0.0027 (0.0003)
Stomach	2.8 (0.4)	1.4 (0.1)	0.055 (0.010)	0.052 (0.009)	0.011 (0.001)	0.0078 (0.0011)
Thyroid	220 (22)	660 (88)	730 (79)	940 (100)	890 (230)	550 (60)

Data is presented as mean (SEM) (n=5). nd indicates that no samples were collected at the correlating time points.

Table S2: ^{131}I activity concentration c_{tissue} in %IA/g over time in male and female rats exposed at an age of 17 (adult) weeks. The activity concentration is decay corrected to time of injection and displayed for 6 measured time points ranging from 1 hour to 144 hours, and a selection of different organs and tissues. The injected activity was on average 0.36 (SEM = 0.05) MBq. It should be noted that all rats in the 1-hour-group were not euthanized exactly 1 h after injection.

	$c_{tissue}(t)$ [%IA/g]					
Tissue	1 hour	6 hours	18 hours	24 hours	72 hours	144 hours
Male, Adult						
Blood	0.19 (0.02)	0.12 (0.01)	0.015 (0.001)	0.013 (0.002)	0.0035 (0.0005)	0.0026 (0.0003)
Bone marrow	0.17 (0.05)	0.060 (0.004)	0.064 (0.036)	0.0073 (0.0008)	0.0055 (0.002)	0.0032 (0.0017)
Brain	0.010 (0.002)	0.0052 (0.0005)	0.0044 (0.0029)	0.0013 (0.0002)	0.0019 (0.0007)	0.00082 (0.00014)
Carcass	nd	0.073 (0.002)	nd	0.012 (0.001)	nd	0.0074 (0.0006)
Gonads	nd	0.060 (0.004)	nd	0.0065 (0.0007)	nd	0.00024 (0.00001)
Heart	0.058 (0.005)	0.037 (0.003)	0.0054 (0.0003)	0.0044 (0.0007)	0.0022 (0.0004)	0.0013 (0.0001)
Kidney	0.11 (0.01)	0.075 (0.010)	0.011 (0.001)	0.012 (0.002)	0.0055 (0.0003)	0.0041 (0.0004)
Large intestine	0.13 (0.05)	0.084 (0.011)	0.0088 (0.0014)	0.0081 (0.0011)	0.0045 (0.0016)	0.0025 (0.0003)
Liver	0.076 (0.008)	0.048 (0.004)	0.0098 (0.0007)	0.011 (0.002)	0.0080 (0.0006)	0.0054 (0.0005)
Lung	0.41 (0.07)	0.069 (0.005)	0.0089 (0.0005)	0.0088 (0.0015)	0.0035 (0.0006)	0.0022 (0.0003)
Muscle	0.030 (0.001)	0.029 (0.005)	0.0034 (0.0005)	0.0026 (0.0004)	0.0012 (0.0003)	0.00067 (0.00008)
Salivary gland	0.077 (0.007)	0.060 (0.004)	0.0075 (0.0006)	0.0063 (0.0009)	0.0026 (0.00069)	0.0015 (0.0001)
Small intestine	0.11 (0.02)	0.077 (0.004)	0.0086 (0.0003)	0.0088 (0.0016)	0.0035 (0.0005)	0.0025 (0.0008)
Spleen	0.087 (0.014)	0.055 (0.004)	0.0062 (0.0004)	0.0061 (0.0009)	0.0016 (0.0001)	0.0013 (0.0003)
Stomach	0.76 (0.08)	0.47 (0.05)	0.041 (0.004)	0.031 (0.003)	0.0036 (0.0003)	0.0042 (0.0017)
Thyroid	79 (18)	310 (20)	320 (29)	310 (59)	150 (19)	110 (13)
Female, Adult						
Blood	0.63 (0.09)	0.35 (0.03)	0.056 (0.009)	0.031 (0.004)	0.018 (0.003)	0.0075 (0.0008)
Bone marrow	0.38 (0.05)	0.22 (0.02)	0.098 (0.052)	0.022 (0.005)	0.0073 (0.0007)	0.0018 (0.0009)
Brain	0.025 (0.003)	0.014 (0.001)	0.0062 (0.0017)	0.0022 (0.0005)	0.0069 (0.0012)	0.0016 (0.0002)
Carcass	nd	0.17 (0.01)	nd	0.025 (0.004)	nd	0.013 (0.001)
Gonads	nd	0.21 (0.01)	nd	0.021 (0.003)	nd	0.0067 (0.0017)
Heart	0.20 (0.02)	0.11 (0.01)	0.020 (0.003)	0.010 (0.002)	0.0082 (0.0013)	0.0029 (0.0003)
Kidney	0.36 (0.04)	0.21 (0.01)	0.036 (0.006)	0.026 (0.005)	0.021 (0.003)	0.0080 (0.0008)
Large intestine	0.32 (0.08)	0.23 (0.01)	0.033 (0.005)	0.018 (0.002)	0.011 (0.002)	0.0042 (0.0005)
Liver	0.31 (0.05)	0.16 (0.01)	0.034 (0.005)	0.030 (0.008)	0.033 (0.005)	0.012 (0.001)
Lung	0.36 (0.05)	0.22 (0.01)	0.038 (0.005)	0.021 (0.003)	0.014 (0.003)	0.0053 (0.0005)
Muscle	0.11 (0.04)	0.074 (0.009)	0.037 (0.024)	0.037 (0.024)	0.028 (0.024)	0.0014 (0.0001)
Salivary gland	0.34 (0.14)	0.17 (0.01)	0.032 (0.01)	0.016 (0.002)	0.015 (0.004)	0.0033 (0.0003)
Small intestine	0.54 (0.22)	0.32 (0.04)	0.038 (0.006)	0.021 (0.002)	0.011 (0.001)	0.0040 (0.0004)
Spleen	0.26 (0.04)	0.17 (0.01)	0.029 (0.005)	0.014 (0.002)	0.0084 (0.0023)	0.0031 (0.0004)
Stomach	1.4 (0.2)	1.2 (0.2)	0.23 (0.04)	0.077 (0.023)	0.023 (0.005)	0.0054 (0.0005)
Thyroid	66 (13)	540 (66)	780 (96)	1100 (350)	770 (97)	470 (47)

Data is presented as the mean value (n=5-6) plus standard error of the mean (SEM). nd indicates that no samples were collected at the correlating time points.
