# Supplementary material for: Sex and age at exposure influence 131I biodistribution and dosimetry in Sprague–Dawley rats
Source: Biol Sex Differ. 2026 Sep 18;17:154. doi: 10.1186/s13293-026-00989-4 (PMC13589867; doi:10.1186/s13293-026-00989-4)
Supplement: Supplementary file 2 [file 13293_2026_989_MOESM3_ESM.pdf]

Additional file 3

Mean absorbed dose per unit injected activity in examined tissues of young and adult male and female rats, calculated using the analytical method.

Mean absorbed dose per unit injected activity, $D/A_{inj}(t)$, expressed as mGy/MBq, calculated for examined organs and tissues of male and female Sprague–Dawley rats exposed to ^{131}I at 5 (young) or 17 (adult) weeks of age. Absorbed doses are presented from time of injection to each termination time point (1 h to 144 h), as well as extrapolated to infinity.

Data are based on measured activity concentrations and were calculated using the analytical method, including contributions from emitted electrons and photons. "nd" indicates that D/A_{inj} was not calculated at the corresponding time point, as the first termination point with tissue collection was at 6 h.

Table S1: **Mean absorbed dose per unit injected activity, $D/A_{inj}(t)$, to examined organs and tissues in male and female rats exposed at an age of 5 (young) weeks, calculated using the analytical method.** The values are given from time of injection to the 6 termination points (from 1 hour to 144 hours) as well as infinity.

	$D/A_{inj}(t)$ [mGy/MBq]						
Tissue	1 hour	6 hours	18 hours	24 hours	72 hours	144 hours	∞
Male, Young							
Blood	4.7	6.8	8.5	8.8	9.9	11	11
Bone marrow	0.23	1.5	2.4	2.6	3.3	3.8	4.1
Brain	0.081	0.40	0.75	0.84	1.3	1.7	2.2
Carcass	nd	1.0	2.7	3.1	4.5	5.7	6.8
Gonads	0.39	2.2	3.0	3.1	3.8	4.4	5.1
Heart	0.18	1.1	1.8	2.0	2.7	3.2	3.6
Kidney	0.30	1.9	3.0	3.3	4.3	5.1	5.7
Large Intestine	0.25	1.7	2.9	3.2	4.1	4.7	5.1
Liver	0.20	1.3	2.3	2.6	3.6	4.5	5.2
Lungs	0.23	1.5	2.8	3.0	3.9	4.6	5.0
Muscle	0.15	0.91	1.6	1.8	2.4	2.9	3.3
Salivary gland	0.25	1.6	2.7	2.9	3.7	4.3	4.8
Small intestine	0.29	2.1	4.2	4.7	6.0	6.5	6.9
Spleen	0.22	1.4	2.4	2.6	3.3	3.7	4.1
Stomach	1.3	9.6	18	19	22	23	24
Thyroid	59	1100	6000	8600	25000	40000	56000
Female, Young							
Blood	5.7	8.1	10	10	11	12	12
Bone marrow	0.29	1.9	3.7	4.2	5.8	6.5	7.2
Brain	0.093	0.45	0.87	1.0	1.9	2.5	3.3
Carcass	nd	1.1	2.7	3.1	6.1	8.7	12
Gonads	0.35	2.3	4.0	4.4	6.3	7.2	7.9
Heart	0.22	1.3	2.2	2.4	3.4	4.1	4.9
Kidney	0.34	2.1	3.4	3.7	5.1	6.2	7.2
Large Intestine	0.38	2.3	3.6	3.9	5.1	5.9	6.7
Liver	0.25	1.6	2.9	3.2	4.9	6.2	7.5
Lungs	0.28	1.8	3.2	3.5	4.7	5.6	6.5
Muscle	0.18	1.0	1.9	2.2	3.3	4.0	4.7
Salivary gland	0.29	1.9	3.2	3.5	4.6	5.5	6.4
Small intestine	0.36	2.5	4.7	5.2	6.7	7.7	8.6
Spleen	0.25	1.6	2.8	3.0	4.0	4.7	5.4
Stomach	1.8	13	21	22	24	25	26
Thyroid	88	1700	7800	11000	38000	65000	100000

nd indicates that D/A_{inj} was not calculated at this time point, as the first termination point with tissue collection was at 6 h.

Table S2: Mean absorbed dose per unit injected activity, $D/A_{inj}(t)$, to examined organs and tissues in male and female rats exposed at an age of 17 (adult) weeks, calculated using the analytical method. The absorbed doses are displayed for the 6 termination points ranging from 1 hour to 144 hours, as well as infinity.

	$D/A_{inj}(t)$ [mGy/MBq]						
Tissue	1 hour	6 hours	18 hours	24 hours	72 hours	144 hours	∞
Male, Adult							
Blood	3.6	4.3	5.1	5.3	5.8	6.1	6.3
Bone marrow	0.28	0.81	1.3	1.4	1.8	2.2	2.6
Brain	0.075	0.20	0.38	0.43	0.68	0.90	1.2
Carcass	nd	0.31	0.82	1.0	1.5	2.1	3.1
Gonads	nd	0.26	0.67	0.85	1.0	1.3	1.5
Heart	0.14	0.41	0.78	0.86	1.2	1.4	1.6
Kidney	0.22	0.67	1.3	1.4	2.0	2.4	2.7
Large Intestine	0.24	0.74	1.4	1.5	2.0	2.3	2.6
Liver	0.16	0.49	0.95	1.1	1.7	2.2	2.7
Lungs	0.60	1.4	1.8	1.9	2.4	2.7	2.9
Muscle	0.10	0.29	0.58	0.65	0.93	1.1	1.3
Salivary gland	0.17	0.51	1.0	1.1	1.6	1.8	2.0
Small intestine	0.21	0.65	1.3	1.4	1.9	2.2	2.4
Spleen	0.18	0.54	1.0	1.1	1.5	1.7	1.9
Stomach	1.1	3.5	6.4	6.9	7.8	8.1	8.2
Thyroid	73	620	3500	4700	11000	16000	23000
Female, Adult							
Blood	4.3	6.7	9.1	9.5	11	12	12
Bone marrow	0.36	2.0	4.0	4.4	5.7	6.2	6.7
Brain	0.071	0.36	0.73	0.84	1.4	2.0	3.2
Carcass	nd	0.80	1.8	2.1	3.5	4.8	6.1
Gonads	nd	0.93	2.0	2.2	3.4	4.3	5.0
Heart	0.21	1.1	2.1	2.3	3.1	3.8	4.4
Kidney	0.35	1.9	3.5	3.9	5.4	6.5	7.6
Large Intestine	0.32	1.8	3.6	4.0	5.3	6.0	6.7
Liver	0.30	1.6	2.9	3.2	4.9	6.5	8.4
Lungs	0.35	1.9	3.6	4	5.3	6.2	6.9
Muscle	0.15	0.79	1.6	1.7	2.4	2.9	3.4
Salivary gland	0.33	1.7	3.1	3.3	4.4	5.3	6.1
Small intestine	0.50	2.7	5.0	5.4	6.7	7.4	8.0
Spleen	0.27	1.5	2.8	3.1	4.2	4.8	5.4
Stomach	1.3	7.6	16	18	21	22	23
Thyroid	27	1100	7000	11000	39000	62000	83000

nd indicates that D/A_{inj} was not calculated at this time point, as the first termination point with tissue collection was at 6 h.
