# Supplementary material for: Sex and age at exposure influence 131I biodistribution and dosimetry in Sprague–Dawley rats
Source: Biol Sex Differ. 2026 Sep 18;17:154. doi: 10.1186/s13293-026-00989-4 (PMC13589867; doi:10.1186/s13293-026-00989-4)
Supplement: Supplementary file 3 [file 13293_2026_989_MOESM4_ESM.pdf]

Additional file 4

Mean absorbed dose per unit injected activity in examined tissues of young and adult male and female rats, calculated using the Monte Carlo (MC) method.

Mean absorbed dose per unit injected activity, $D/A_{inj}(t)$, expressed as mGy/MBq, calculated for examined organs and tissues of male and female Sprague–Dawley rats exposed to ^{131}I at 5 (young) or 17 (adult) weeks of age. Absorbed doses are presented from time of injection to each termination time point (1 h to 144 h), as well as extrapolated to infinity.

Dose calculations were based on the measured activity concentrations and performed according to the MIRD formalism using MC-derived absorbed fractions. Group-specific voxel-based ROBY phantoms were used to model radiation transport and energy deposition. The absorbed dose to each target organ was calculated by summing contributions from all simulated source regions and included contribution from photons and electrons emitted by ^{131}I.

Blood, muscle, and salivary gland were not represented as dedicated source or target regions in the phantom and were therefore excluded from the Monte Carlo dose calculations. Carcass was included as a structure in the simulation, but no absorbed dose was calculated. "nd" indicates that D/A_{inj} was not calculated at the corresponding time point.

Table S1: **Mean absorbed dose per unit injected activity, $D/A_{inj}(t)$, to examined organs and tissues in male and female rats exposed at an age of 5 (young) weeks, calculated using the Monte Carlo method.** The values are given from time of injection to the 6 termination points (from 1 hour to 144 h) as well as infinity.

	$D/A_{inj}(t)$ [mGy/MBq]						
Tissue	1 hour	6 hours	18 hours	24 hours	72 hours	144 hours	∞
Male, Young							
Blood	nd	nd	nd	nd	nd	nd	nd
Bone marrow	0.0036	0.15	0.45	0.53	0.94	1.3	1.6
Brain	0.0061	0.10	0.29	0.36	0.79	1.2	1.6
Carcass	nd	nd	nd	nd	nd	nd	nd
Gonads	0.36	2.2	3.0	3.1	3.6	4.0	4.4
Heart	0.12	0.91	1.6	1.8	2.3	2.7	2.9
Kidney	0.30	2.1	3.5	3.7	4.8	5.5	6.0
Large Intestine	0.29	2.3	4.1	4.5	5.5	6.0	6.5
Liver	0.15	1.2	2.2	2.5	3.4	4.0	4.5
Lung	0.13	1.1	2.2	2.4	3.0	3.5	3.8
Muscle	nd	nd	nd	nd	nd	nd	nd
Salivary gland	nd	nd	nd	nd	nd	nd	nd
Small intestine	0.34	2.9	6.0	6.7	8.1	8.6	8.9
Spleen	0.19	1.5	2.7	2.9	3.4	3.6	3.8
Stomach wall	2.0	16	30	32	37	38	39
Thyroid	49	950	5000	7200	21000	34000	47000
Female, Young							
Blood	nd	nd	nd	nd	nd	nd	nd
Bone marrow	0.0038	0.13	0.38	0.47	1.1	1.7	2.5
Brain	0.0068	0.10	0.30	0.39	1.0	1.6	2.5
Carcass	nd	nd	nd	nd	nd	nd	nd
Gonads	0.21	1.6	2.9	3.3	4.6	5.2	5.7
Heart	0.11	0.87	1.5	1.6	2.3	2.8	3.4
Kidney	0.36	2.6	4.1	4.4	5.9	6.9	7.7
Large Intestine	0.42	3.0	4.6	4.8	6.0	6.7	7.3
Liver	0.19	1.5	2.6	2.9	4.3	5.3	6.2
Lung	0.13	1.0	1.9	2.0	2.8	3.4	3.9
Muscle	nd	nd	nd	nd	nd	nd	nd
Salivary gland	nd	nd	nd	nd	nd	nd	nd
Small intestine	0.36	3.0	5.5	6.1	7.6	8.4	9.0
Spleen	0.22	1.7	2.9	3.1	3.8	4.2	4.4
Stomach wall	2.4	18	29	30	33	34	34
Thyroid	77	1500	6900	9900	34000	57000	90000

nd indicates that D/A_{inj} was not calculated for this time point

Table S2: Mean absorbed dose per unit injected activity, D/A$_{inj}$(t), to examined organs and tissues in male and female rats exposed at an age of 17 (adult) weeks, calculated using the Monte Carlo method. The absorbed doses are displayed for the 6 termination points ranging from 1 hour to 144 h, as well as infinity.

	D/A$_{inj}$(t) [mGy/MBq]						
Tissue	1 hour	6 hours	18 hours	24 hours	72 hours	144 hours	∞
Male, Adult							
Blood	nd	nd	nd	nd	nd	nd	nd
Bone marrow	0.0057	0.13	0.40	0.46	0.81	1.1	1.6
Brain	0.0084	0.078	0.26	0.32	0.61	0.87	1.3
Carcass	nd	nd	nd	nd	nd	nd	nd
Gonads	nd	0.31	0.83	0.91	1.2	1.5	1.7
Heart	0.17	0.66	1.3	1.5	2.0	2.4	2.8
Kidney	0.18	0.70	1.4	1.6	2.3	2.7	3.1
Large Intestine	0.32	1.2	2.4	2.6	3.5	4.0	4.6
Liver	0.14	0.54	1.1	1.2	2.0	2.5	3.2
Lung	0.87	2.2	2.9	3.0	3.7	4.2	4.7
Muscle	nd	nd	nd	nd	nd	nd	nd
Salivary gland	nd	nd	nd	nd	nd	nd	nd
Small intestine	0.25	0.94	1.9	2.2	2.9	3.3	3.7
Spleen	0.16	0.60	1.2	1.3	1.7	1.9	2.1
Stomach wall	2.5	8.4	15	17	19	19	20
Thyroid	68	580	3300	4400	10000	15000	21000
Female, Adult							
Blood	nd	nd	nd	nd	nd	nd	nd
Bone marrow	0.0044	0.16	0.44	0.54	1.2	1.7	2.2
Brain	0.0090	0.11	0.34	0.44	1.1	1.8	2.6
Carcass	nd	nd	nd	nd	nd	nd	nd
Gonads	nd	0.71	1.6	1.8	2.6	3.3	3.9
Heart	0.19	1.1	2.1	2.3	3.2	3.8	4.4
Kidney	0.39	2.3	4.3	4.7	6.4	7.7	8.8
Large Intestine	0.40	2.5	5.1	5.6	7.2	8.1	8.7
Liver	0.28	1.6	2.9	3.2	4.8	6.3	8.2
Lung	0.27	1.6	3.1	3.4	4.5	5.3	5.9
Muscle	nd	nd	nd	nd	nd	nd	nd
Salivary gland	nd	nd	nd	nd	nd	nd	nd
Small intestine	0.61	3.5	6.5	7.0	8.4	9.2	9.7
Spleen	0.26	1.6	3.2	3.5	4.4	4.9	5.3
Stomach wall	2.3	14	29	32	38	40	41
Thyroid	25	1000	6300	9700	35000	55000	74000

nd indicates that *D/A$_{inj}$* was not calculated for this time point
