# Supplementary material for: Sex and age at exposure influence 131I biodistribution and dosimetry in Sprague–Dawley rats
Source: Biol Sex Differ. 2026 Sep 18;17:154. doi: 10.1186/s13293-026-00989-4 (PMC13589867; doi:10.1186/s13293-026-00989-4)
Supplement: Supplementary file 4 [file 13293_2026_989_MOESM5_ESM.pdf]

Additional file 5

Biokinetic curves fitted to ^{131}I activity concentrations in examined tissues.

Figures showing the fitted biexponential biokinetic curves together with the measured activity concentrations decay corrected to the time of euthanasia, which were used for biokinetic curve fitting and the estimation of the time-integrated activity concentration for absorbed dose calculations.

The y-axis range was adjusted independently for each Figure panel to improve visualization of the measured activity data and the fitted biokinetic curves. Consequently, the panels are intended to illustrate the quality of the curve fits rather than facilitate direct visual comparison between study groups.

In addition to the organ-specific biokinetic curves, the file includes the fitted whole-body activity curves used to estimate the photon contribution to the absorbed dose.

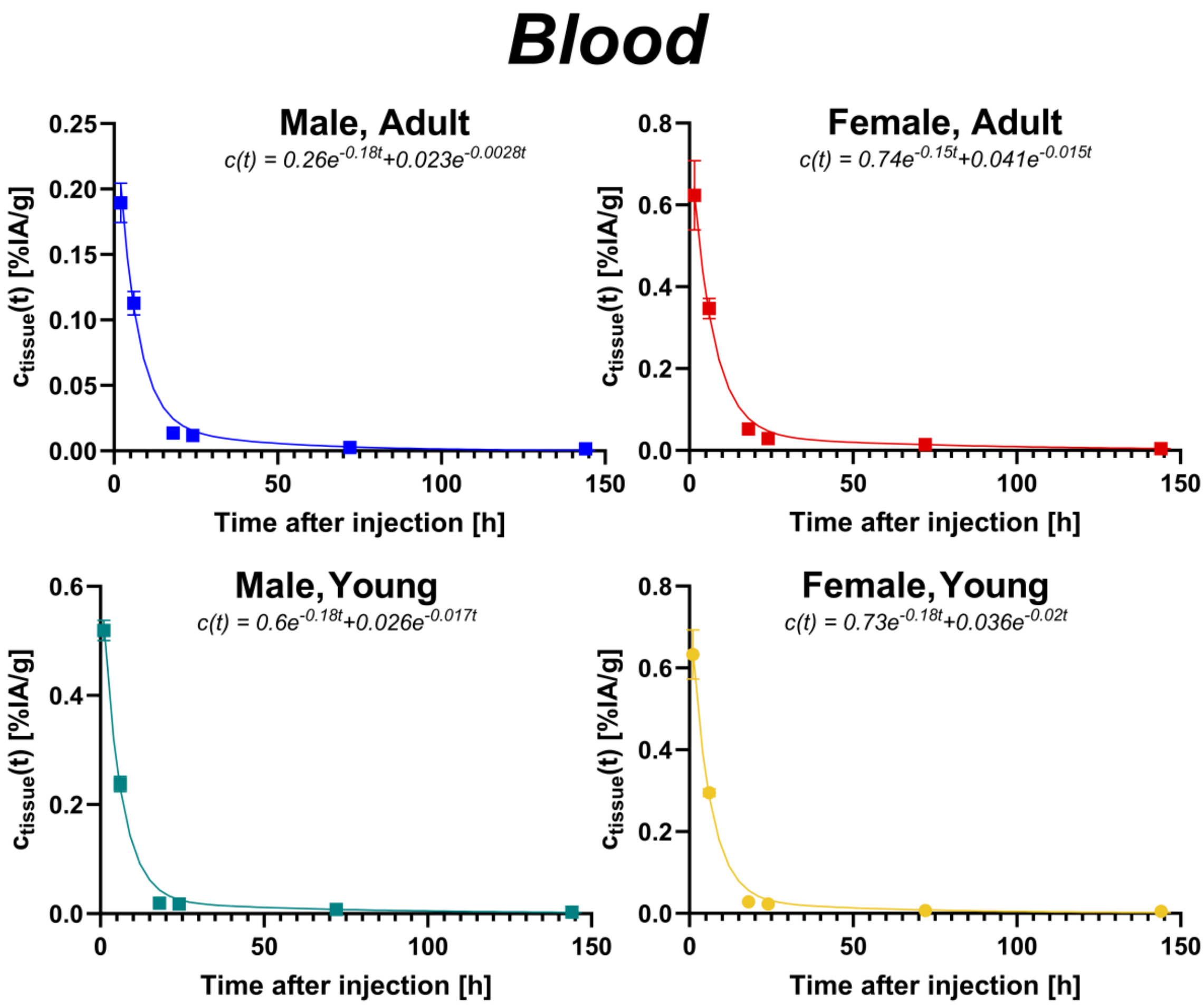

Figure S1: Biokinetic curves for blood. Measured ^{131}I activity concentrations, $c_{\text{tissue}}(t)$, together with the fitted biexponential biokinetic curves for male and female Sprague–Dawley rats exposed at 5 (young) or 17 (adult) weeks of age. The fitted equations used for time-integrated activity concentration estimation are shown in each panel. Data are presented as mean ± SEM. **Note:** The y-axis range differs between panels.

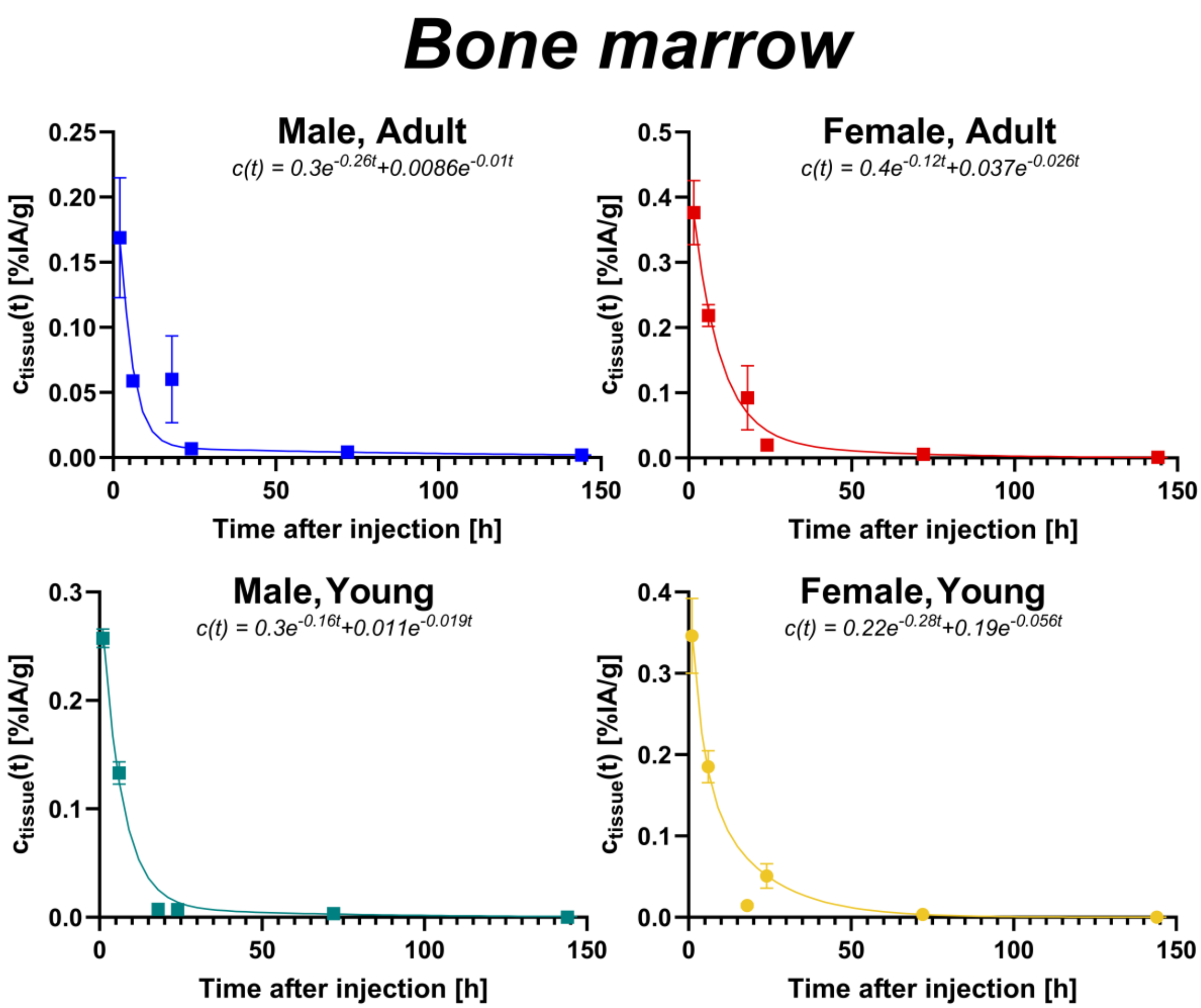

Figure S2: Biokinetic curves for bone marrow. Measured ^{131}I activity concentrations, $c_{\text{tissue}}(t)$, together with the fitted biexponential biokinetic curves for male and female Sprague–Dawley rats exposed at 5 (young) or 17 (adult) weeks of age. The fitted equations used for time-integrated activity concentration estimation are shown in each panel. Data are presented as mean ± SEM. **Note:** The y-axis range differs between panels.

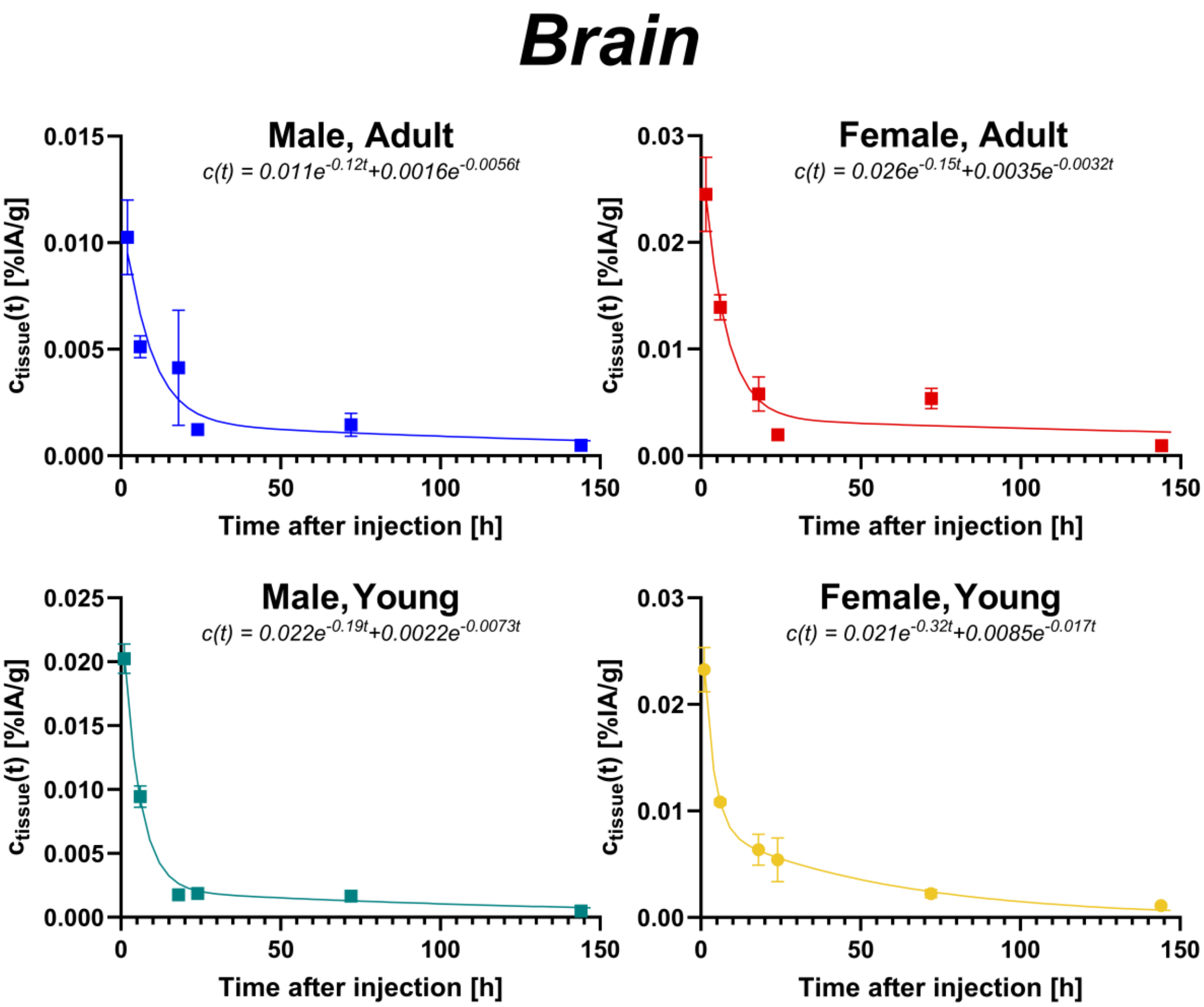

Figure S3: Biokinetic curves for brain. Measured ^{131}I activity concentrations, $c_{\text{tissue}}(t)$, together with the fitted biexponential biokinetic curves for male and female Sprague–Dawley rats exposed at 5 (young) or 17 (adult) weeks of age. The fitted equations used for time-integrated activity concentration estimation are shown in each panel. Data are presented as mean ± SEM. **Note:** The y-axis range differs between panels.

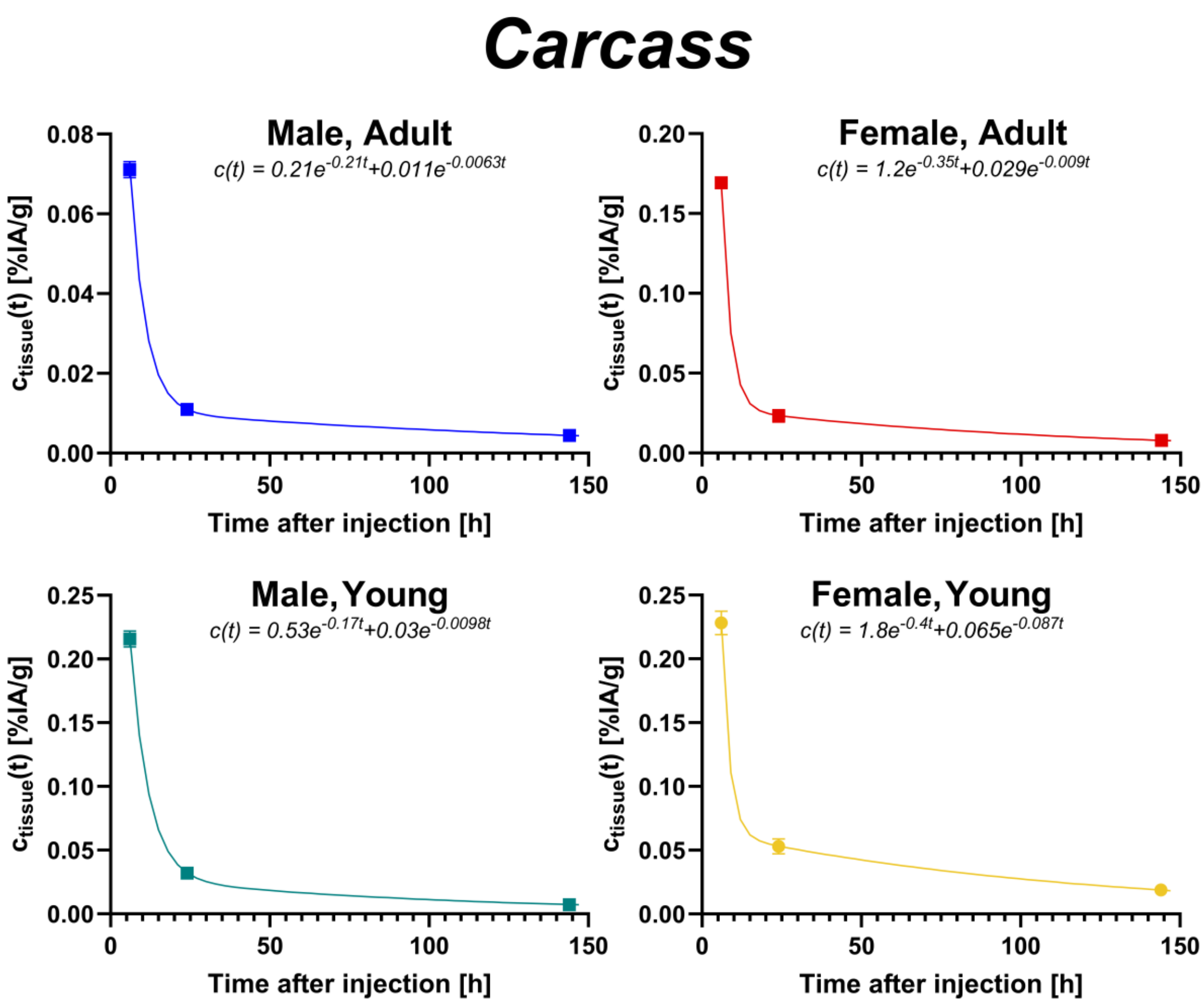

Figure S4: Biokinetic curves for carcass. Measured ^{131}I activity concentrations, $c_{\text{tissue}}(t)$, together with the fitted biexponential biokinetic curves for male and female Sprague–Dawley rats exposed at 5 (young) or 17 (adult) weeks of age. The fitted equations used for time-integrated activity concentration estimation are shown in each panel. Data are presented as mean ± SEM. **Note:** The y-axis range differs between panels.

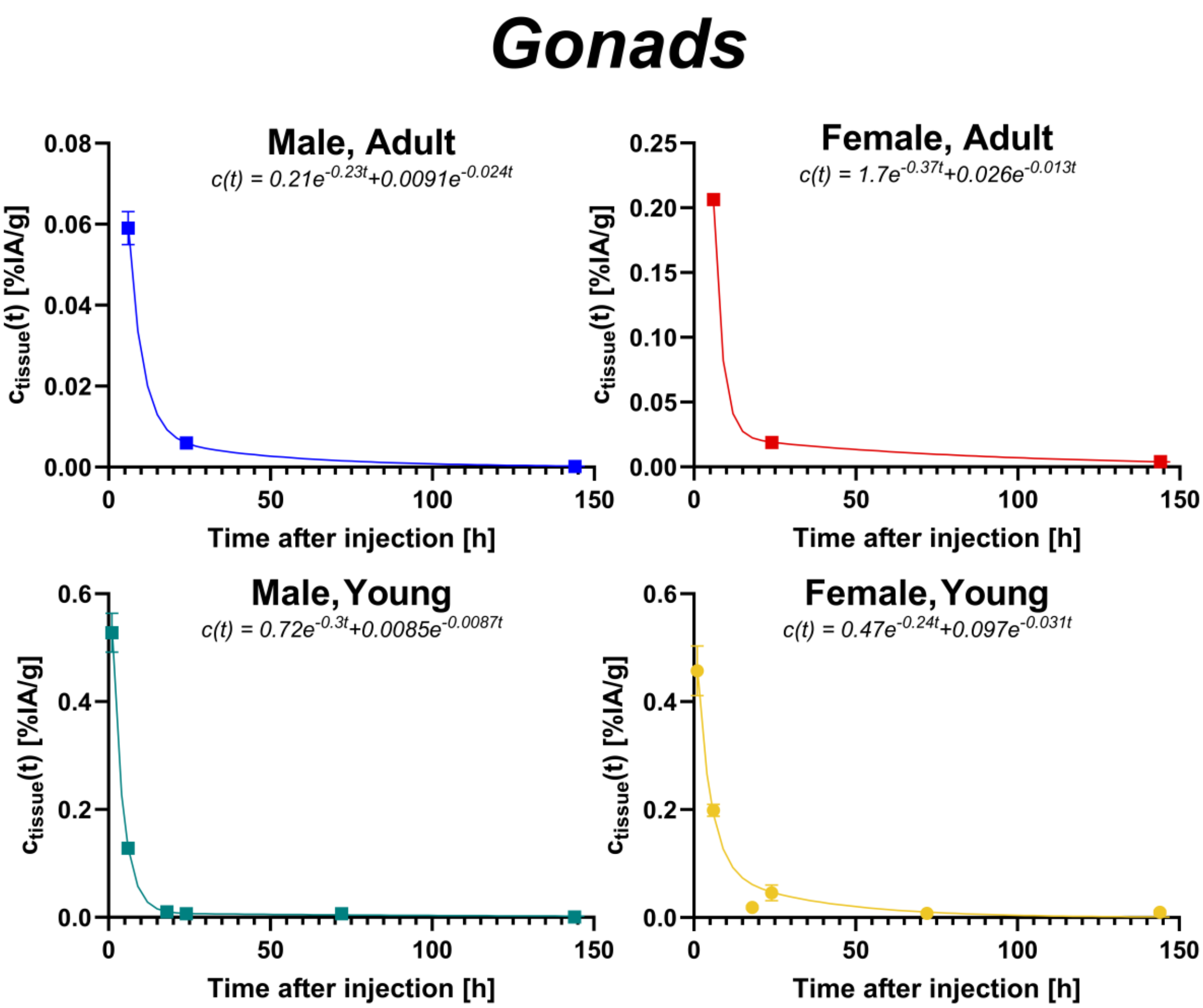

Figure S5: Biokinetic curves for gonads. Measured ^{131}I activity concentrations, $c_{\text{tissue}}(t)$, together with the fitted biexponential biokinetic curves for male and female Sprague–Dawley rats exposed at 5 (young) or 17 (adult) weeks of age. The fitted equations used for time-integrated activity concentration estimation are shown in each panel. Data are presented as mean ± SEM. **Note:** The y-axis range differs between panels.

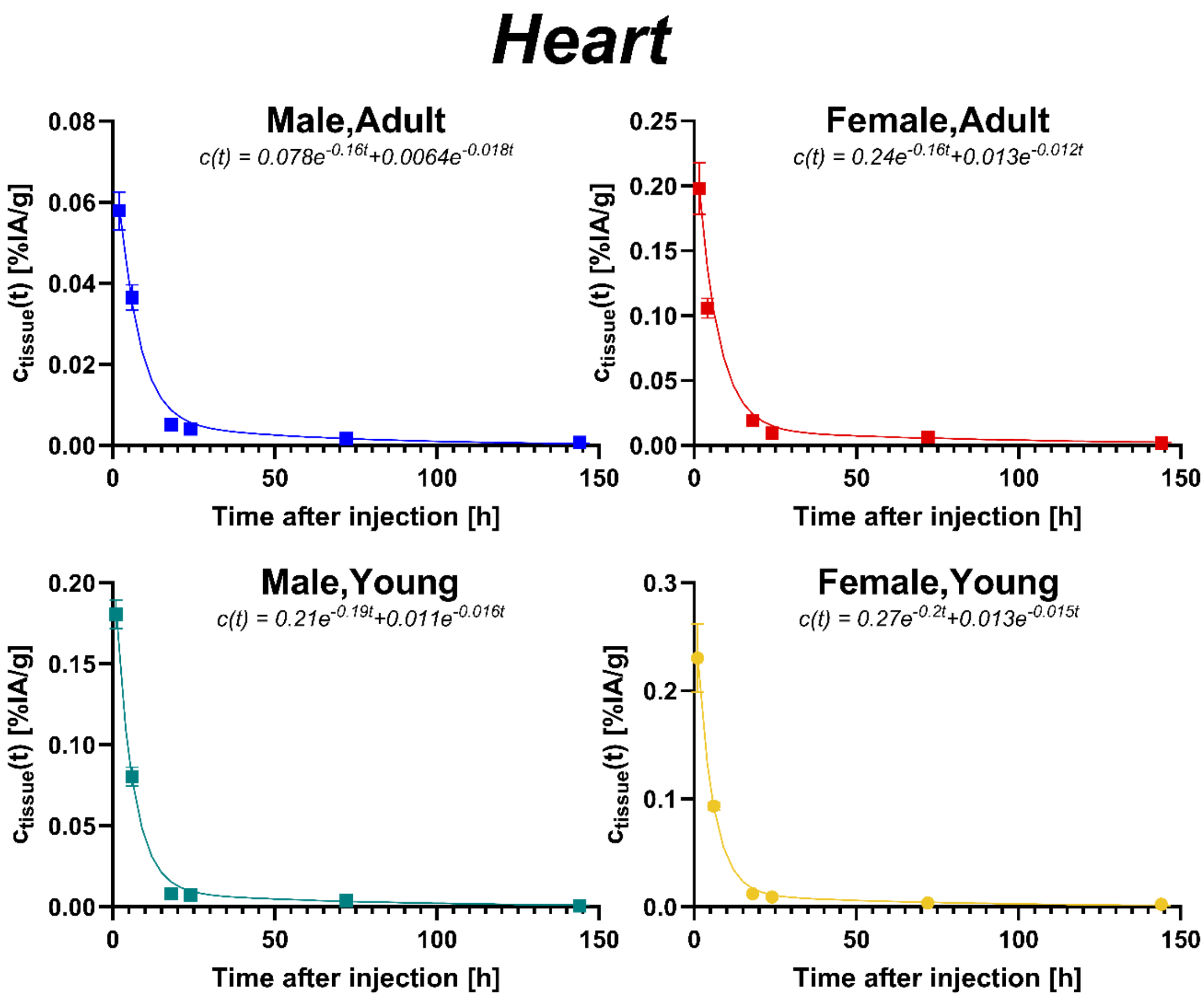

Figure S6: Biokinetic curves for heart. Measured ^{131}I activity concentrations, $c_{\text{tissue}}(t)$, together with the fitted biexponential biokinetic curves for male and female Sprague–Dawley rats exposed at 5 (young) or 17 (adult) weeks of age. The fitted equations used for time-integrated activity concentration estimation are shown in each panel. Data are presented as mean ± SEM. **Note:** The y-axis range differs between panels.

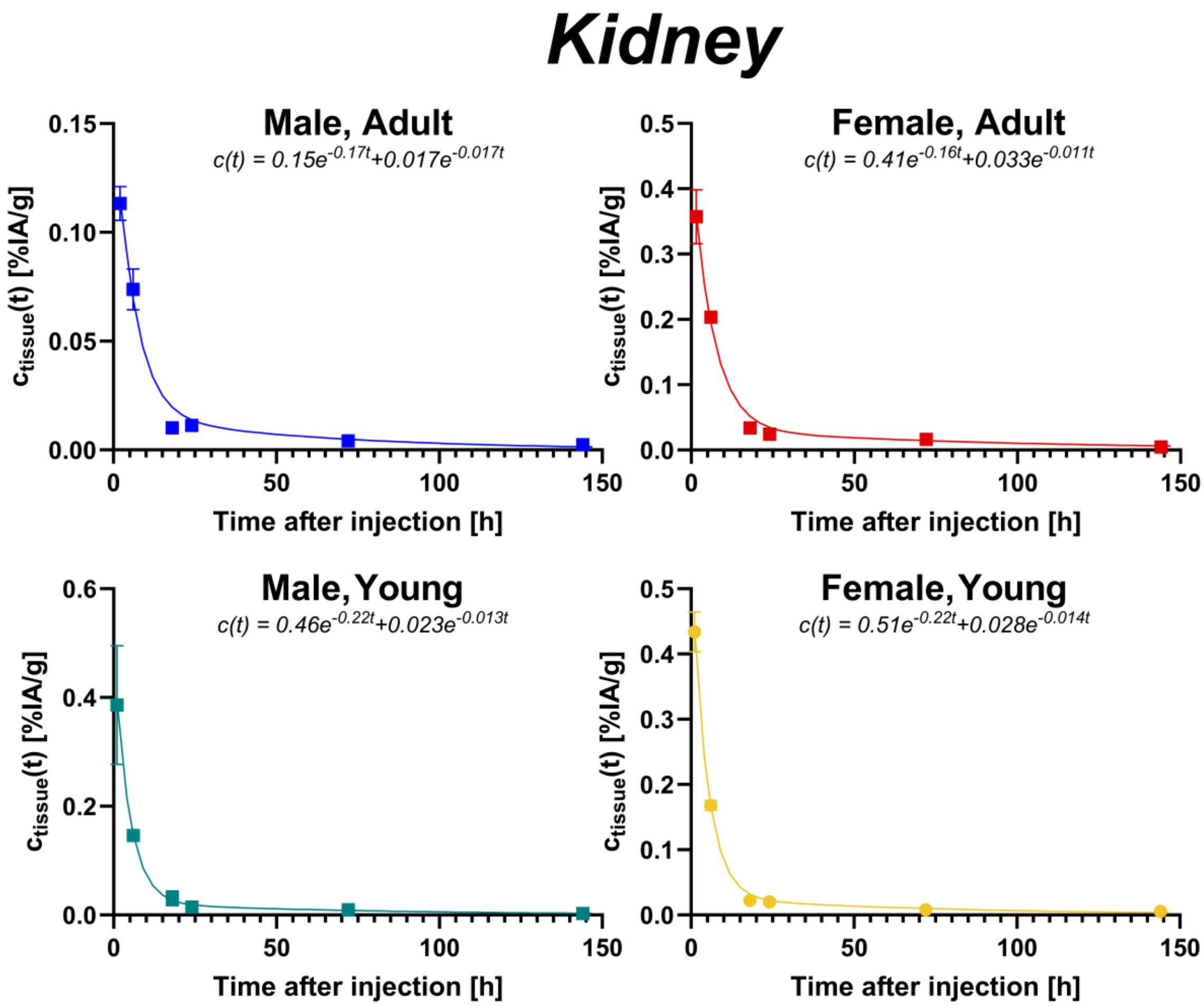

Figure S7: Biokinetic curves for kidney. Measured ^{131}I activity concentrations, $c_{\text{tissue}}(t)$, together with the fitted biexponential biokinetic curves for male and female Sprague–Dawley rats exposed at 5 (young) or 17 (adult) weeks of age. The fitted equations used for time-integrated activity concentration estimation are shown in each panel. Data are presented as mean ± SEM. **Note:** The y-axis range differs between panels.

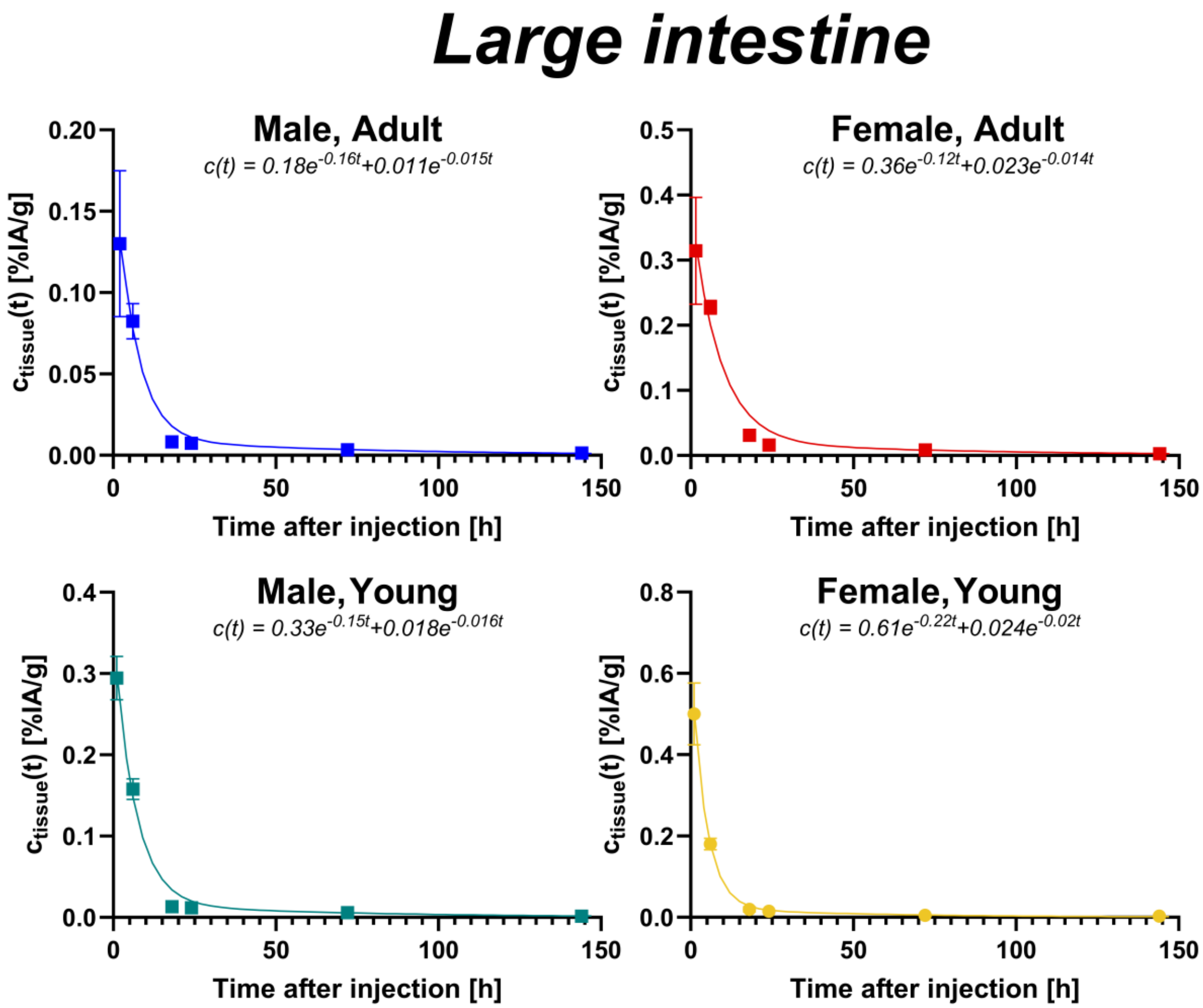

Figure S8: Biokinetic curves for large intestine. Measured ^{131}I activity concentrations, $c_{\text{tissue}}(t)$, together with the fitted biexponential biokinetic curves for male and female Sprague–Dawley rats exposed at 5 (young) or 17 (adult) weeks of age. The fitted equations used for time-integrated activity concentration estimation are shown in each panel. Data are presented as mean ± SEM. **Note:** The y-axis range differs between panels.

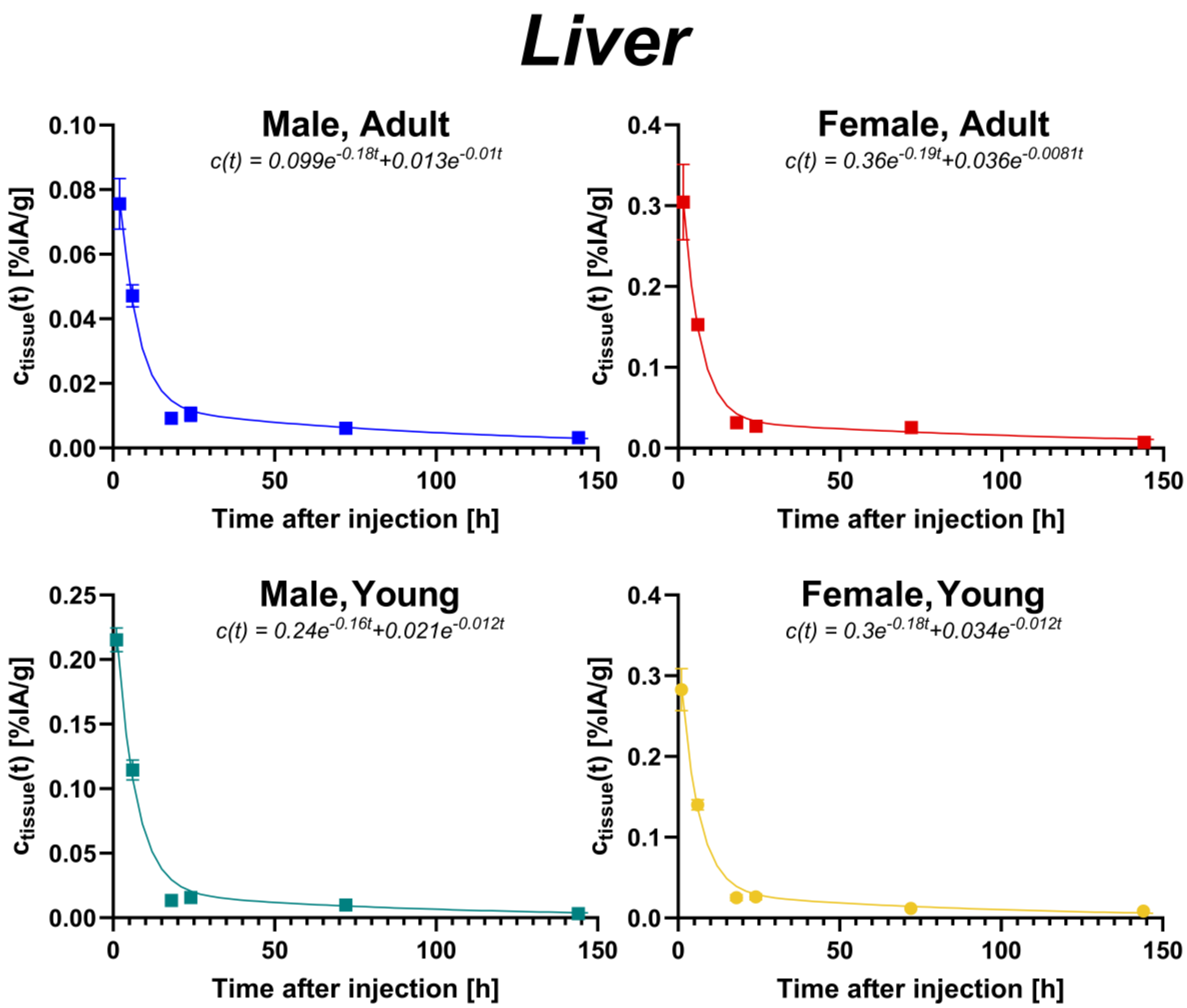

Figure S9: Biokinetic curves for liver. Measured ^{131}I activity concentrations, $c_{\mathrm{tissue}}(t)$, together with the fitted biexponential biokinetic curves for male and female Sprague–Dawley rats exposed at 5 (young) or 17 (adult) weeks of age. The fitted equations used for time-integrated activity concentration estimation are shown in each panel. Data are presented as mean ± SEM. **Note:** The y-axis range differs between panels.

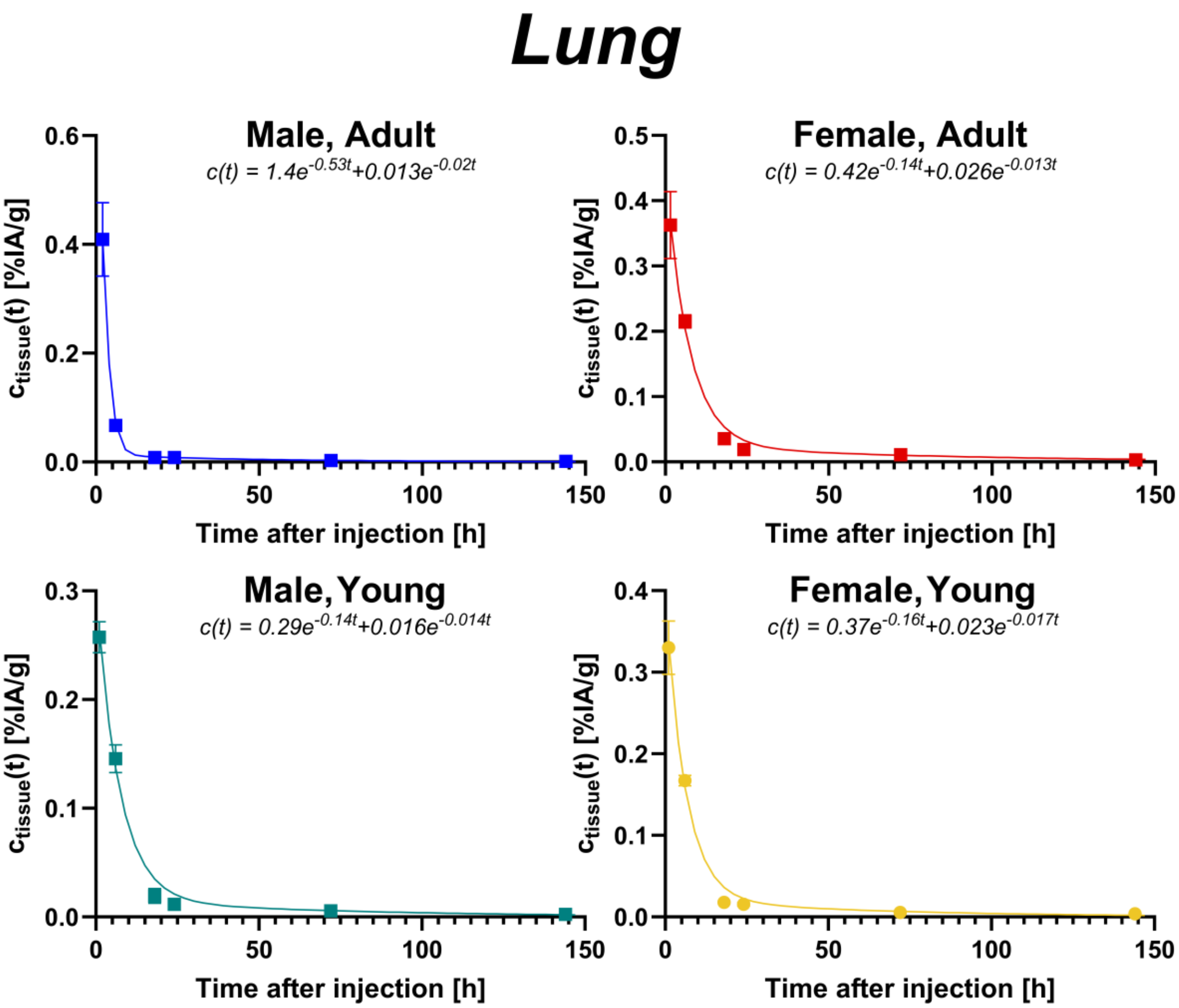

Figure S10: Biokinetic curves for lung. Measured ^{131}I activity concentrations, $c_{\text{tissue}}(t)$, together with the fitted biexponential biokinetic curves for male and female Sprague–Dawley rats exposed at 5 (young) or 17 (adult) weeks of age. The fitted equations used for time-integrated activity concentration estimation are shown in each panel. Data are presented as mean ± SEM. **Note:** The y-axis range differs between panels.

Muscle

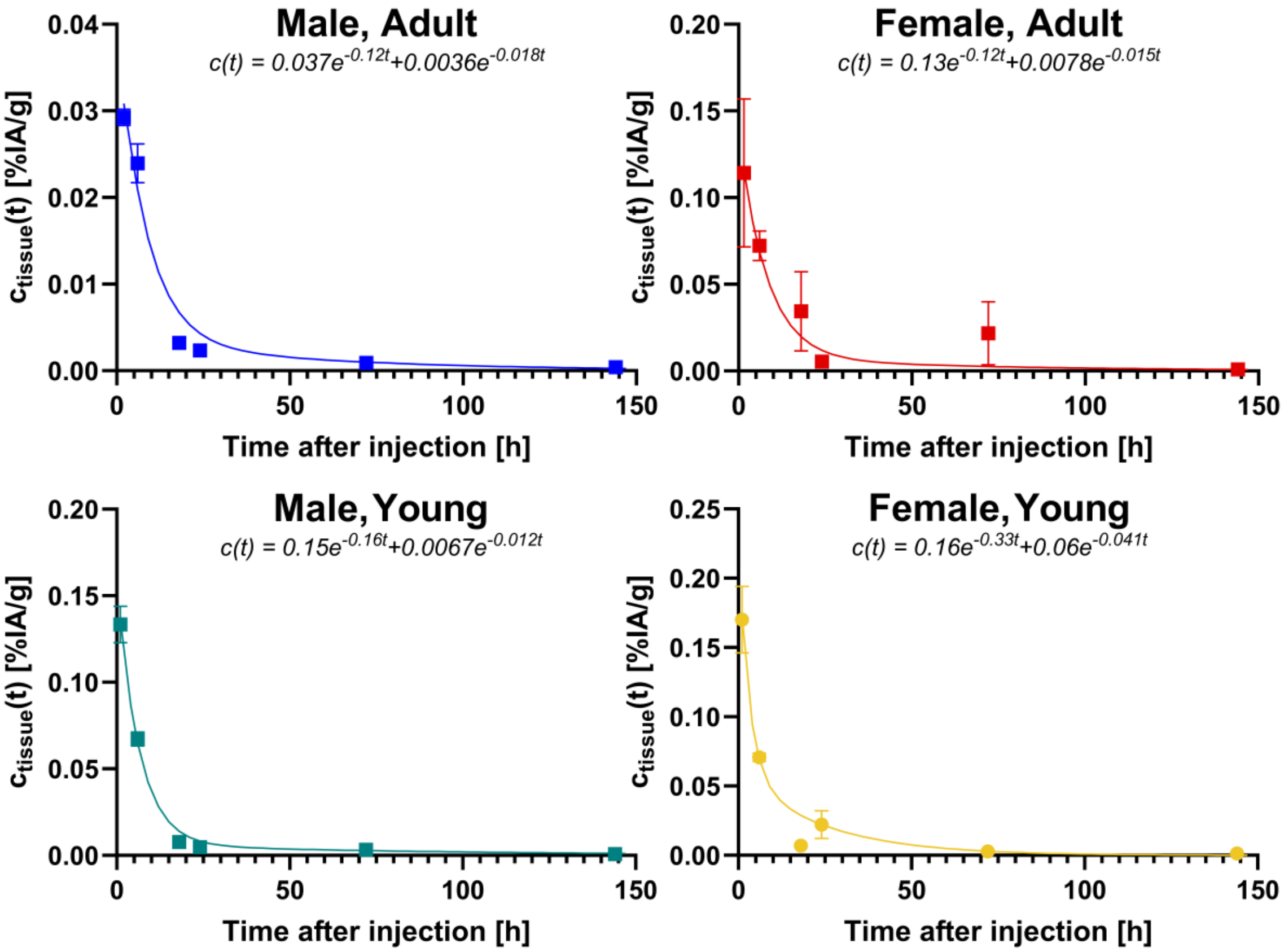

Figure S11: Biokinetic curves for muscle. Measured ^{131}I activity concentrations, $c_{\text{tissue}}(t)$, together with the fitted biexponential biokinetic curves for male and female Sprague–Dawley rats exposed at 5 (young) or 17 (adult) weeks of age. The fitted equations used for time-integrated activity concentration estimation are shown in each panel. Data are presented as mean ± SEM. **Note:** The y-axis range differs between panels.

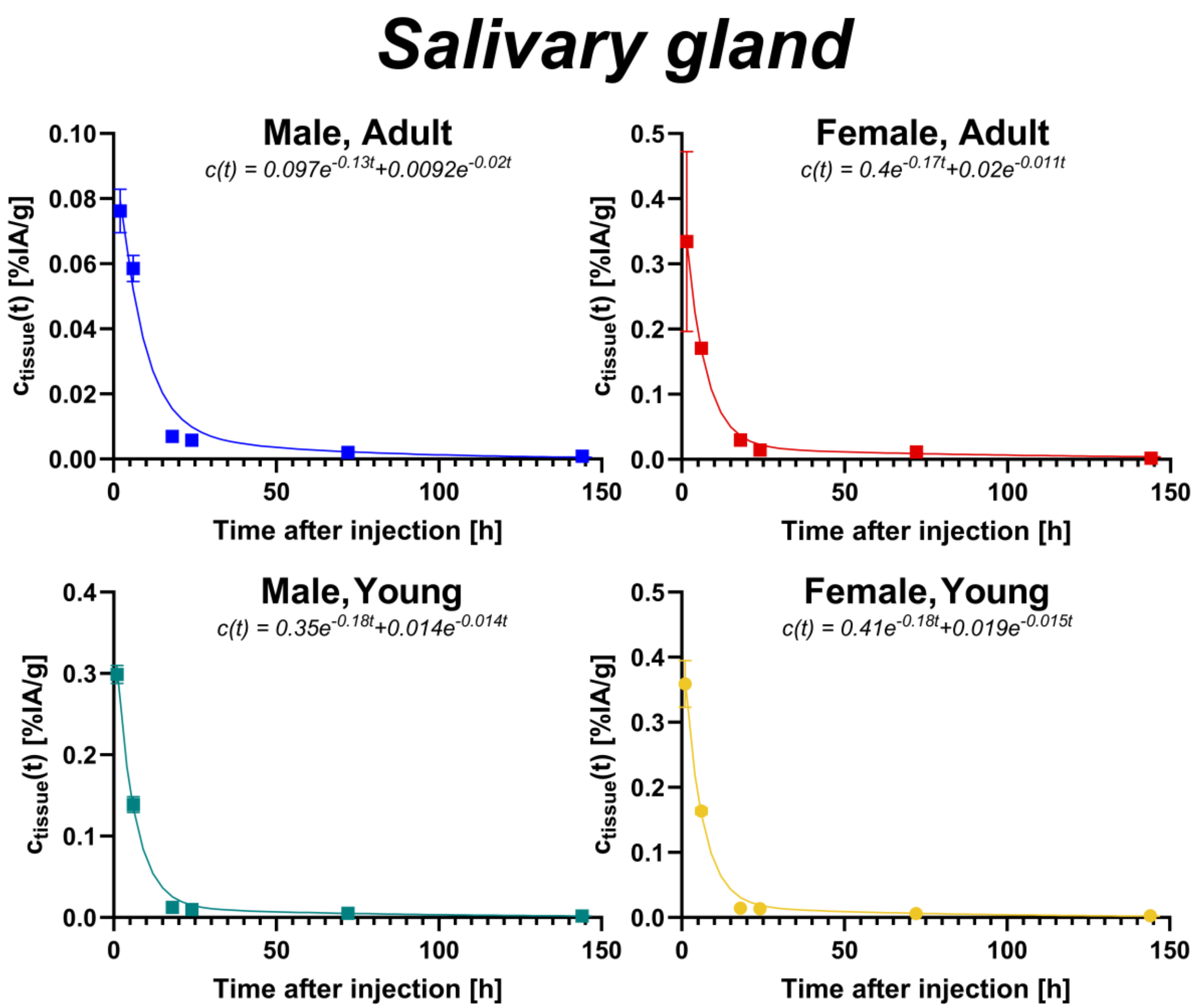

Figure S12: Biokinetic curves for salivary gland. Measured ^{131}I activity concentrations, $c_{\mathrm{tissue}}(t)$, together with the fitted biexponential biokinetic curves for male and female Sprague–Dawley rats exposed at 5 (young) or 17 (adult) weeks of age. The fitted equations used for time-integrated activity concentration estimation are shown in each panel. Data are presented as mean ± SEM. **Note:** The y-axis range differs between panels.

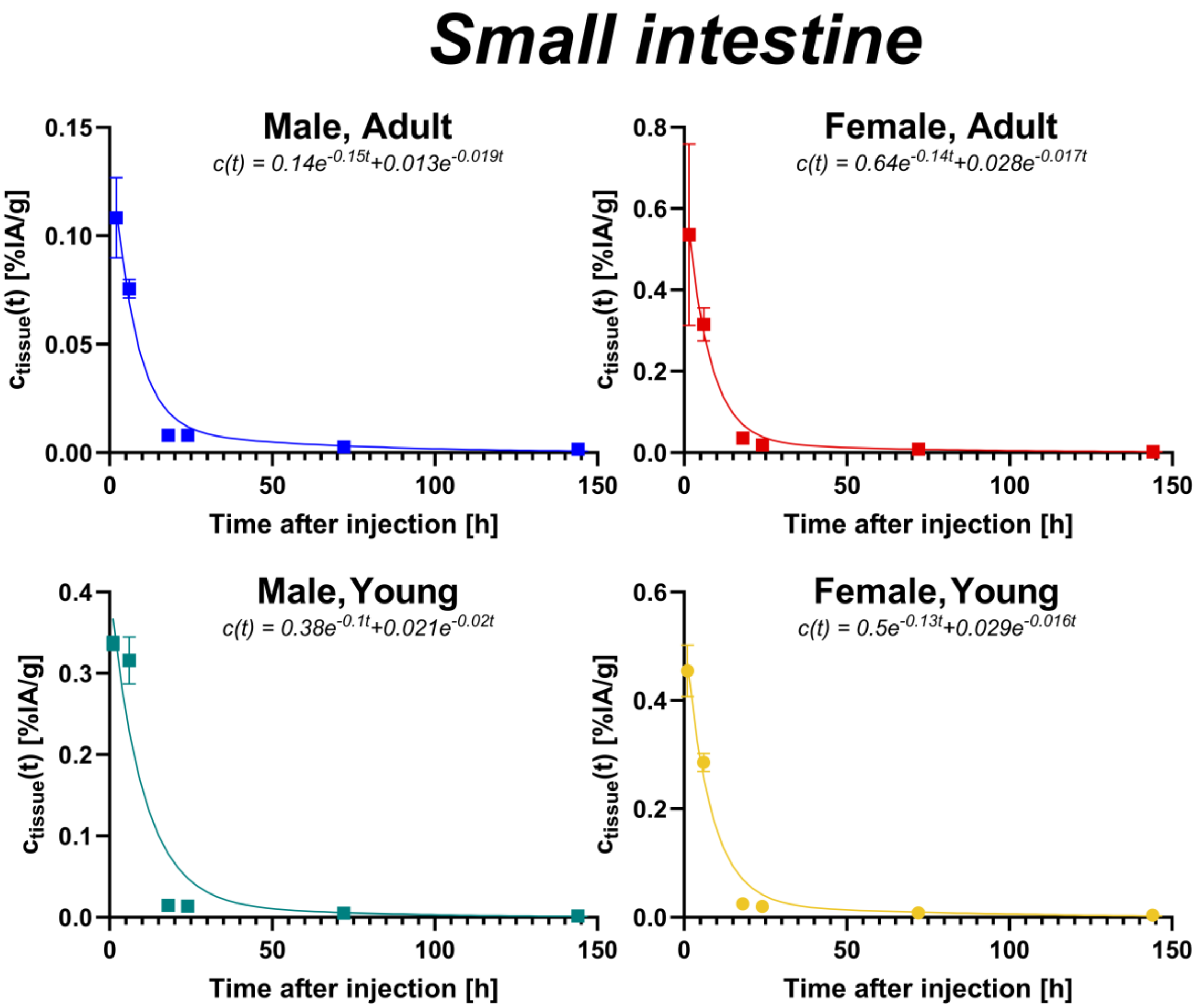

Figure S13: Biokinetic curves for small intestine. Measured ^{131}I activity concentrations, $c_{\text{tissue}}(t)$, together with the fitted biexponential biokinetic curves for male and female Sprague–Dawley rats exposed at 5 (young) or 17 (adult) weeks of age. The fitted equations used for time-integrated activity concentration estimation are shown in each panel. Data are presented as mean ± SEM. **Note:** The y-axis range differs between panels.

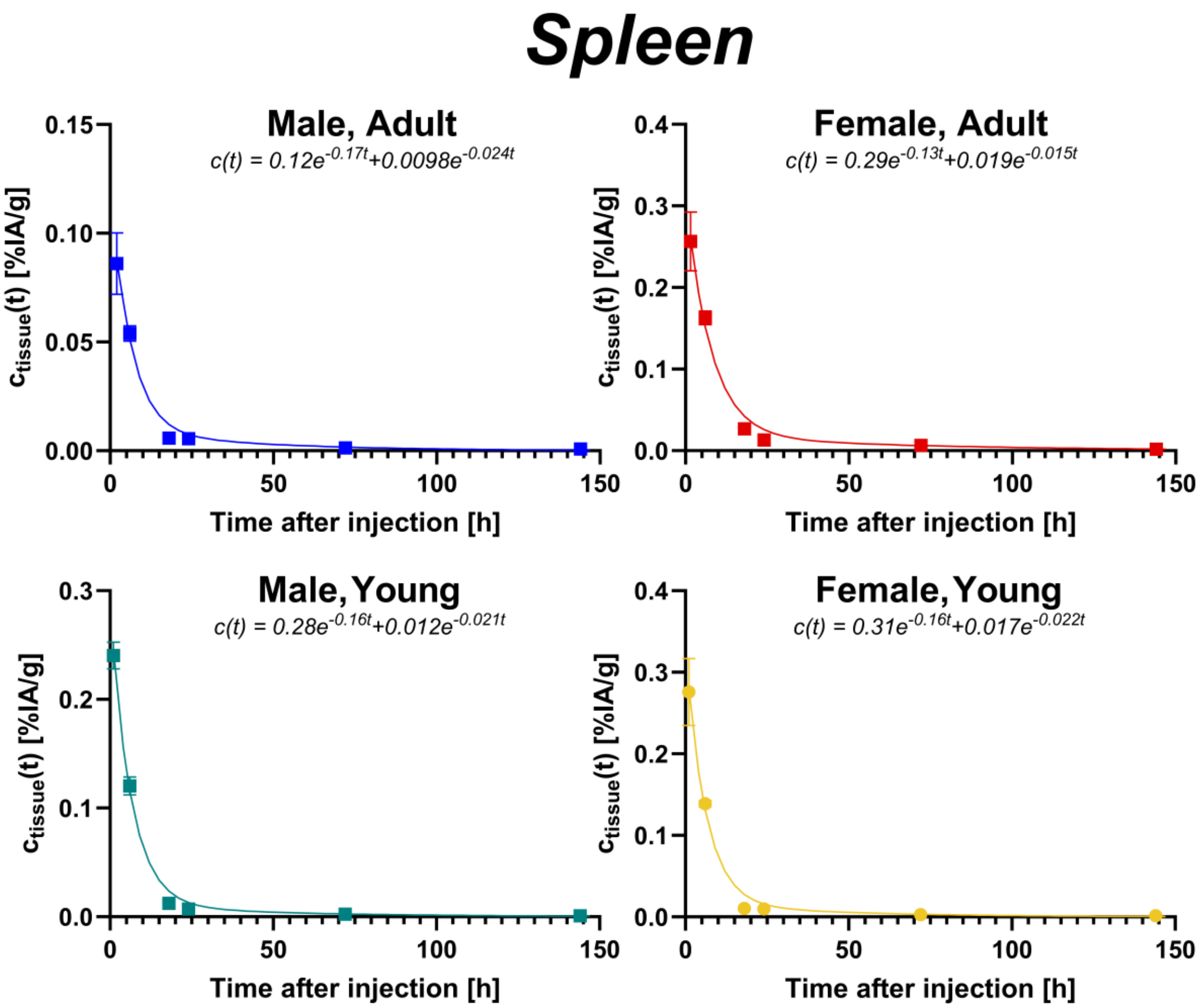

Figure S14: Biokinetic curves for spleen. Measured ^{131}I activity concentrations, $c_{\mathrm{tissue}}(t)$, together with the fitted biexponential biokinetic curves for male and female Sprague–Dawley rats exposed at 5 (young) or 17 (adult) weeks of age. The fitted equations used for time-integrated activity concentration estimation are shown in each panel. Data are presented as mean ± SEM. **Note:** The y-axis range differs between panels.

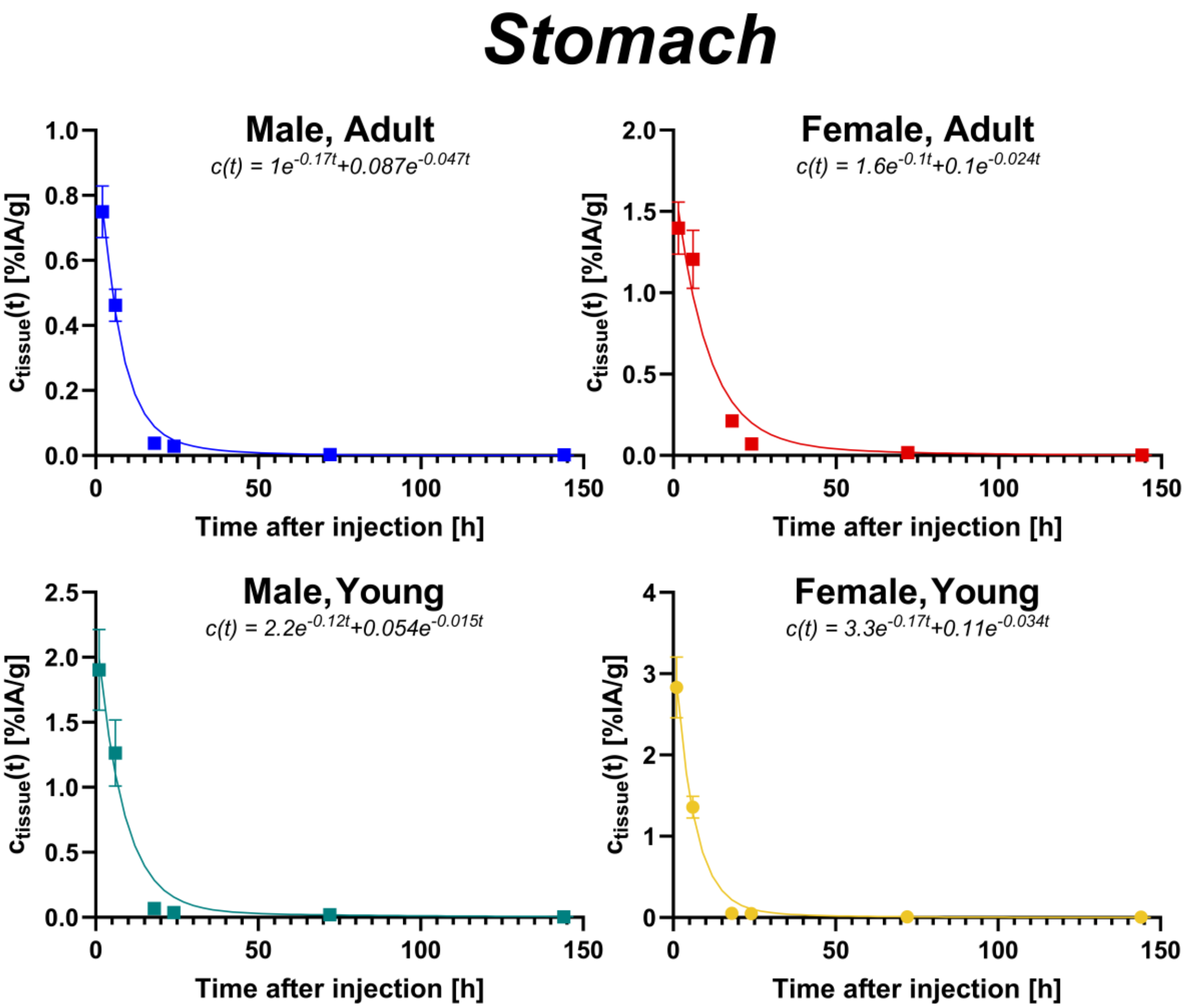

Figure S15: Biokinetic curves for stomach. Measured ^{131}I activity concentrations, $c_{\text{tissue}}(t)$, together with the fitted biexponential biokinetic curves for male and female Sprague–Dawley rats exposed at 5 (young) or 17 (adult) weeks of age. The fitted equations used for time-integrated activity concentration estimation are shown in each panel. Data are presented as mean ± SEM. **Note:** The y-axis range differs between panels.

Thyroid

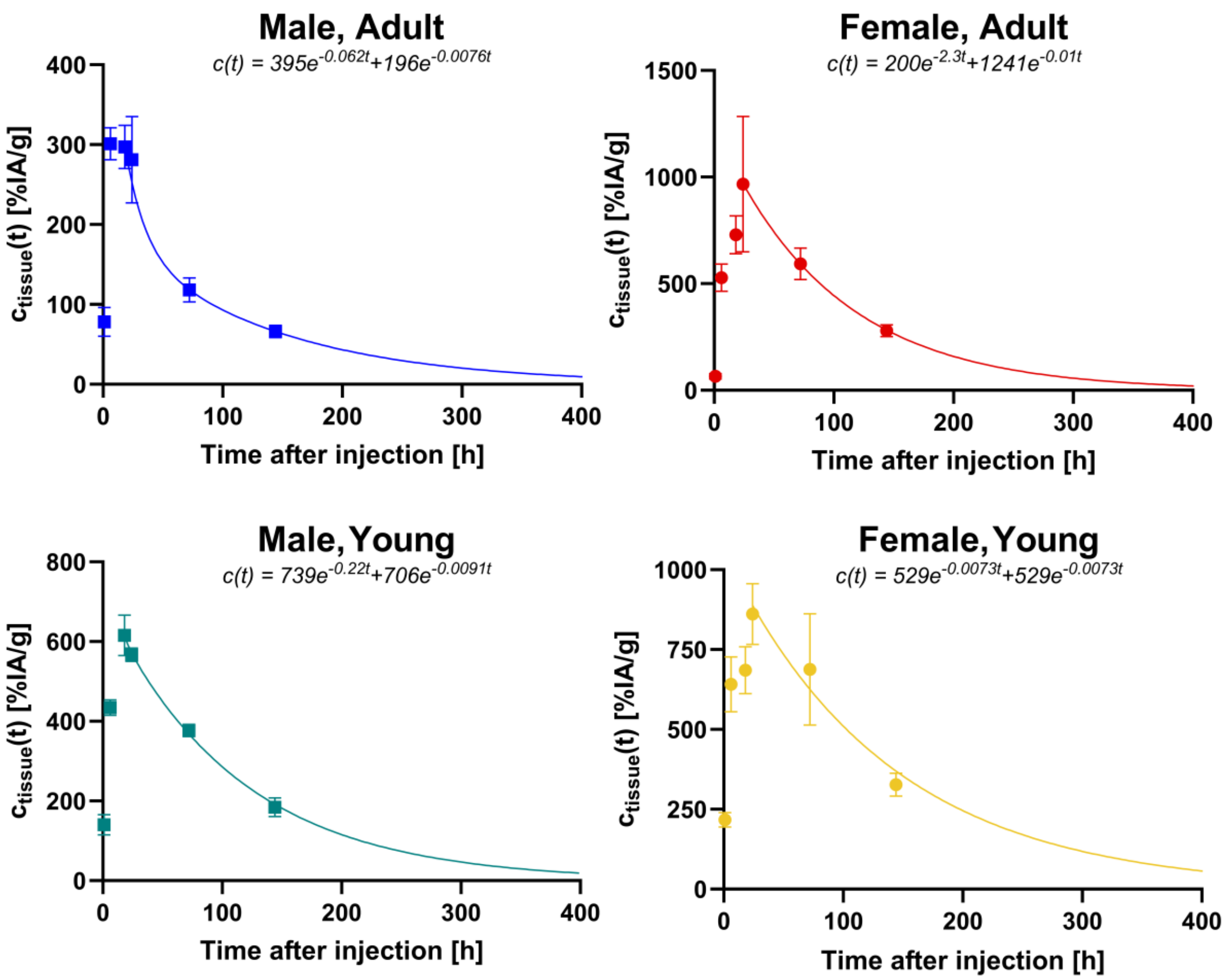

Figure S16: Biokinetic curves for thyroid. Measured ^{131}I activity concentrations, $c_{\text{tissue}}(t)$, together with the fitted biexponential biokinetic curves for male and female Sprague–Dawley rats exposed at 5 (young) or 17 (adult) weeks of age. The fitted equations used for time-integrated activity concentration estimation are shown in each panel. Data are presented as mean ± SEM. **Note:** The y-axis range differs between panels.

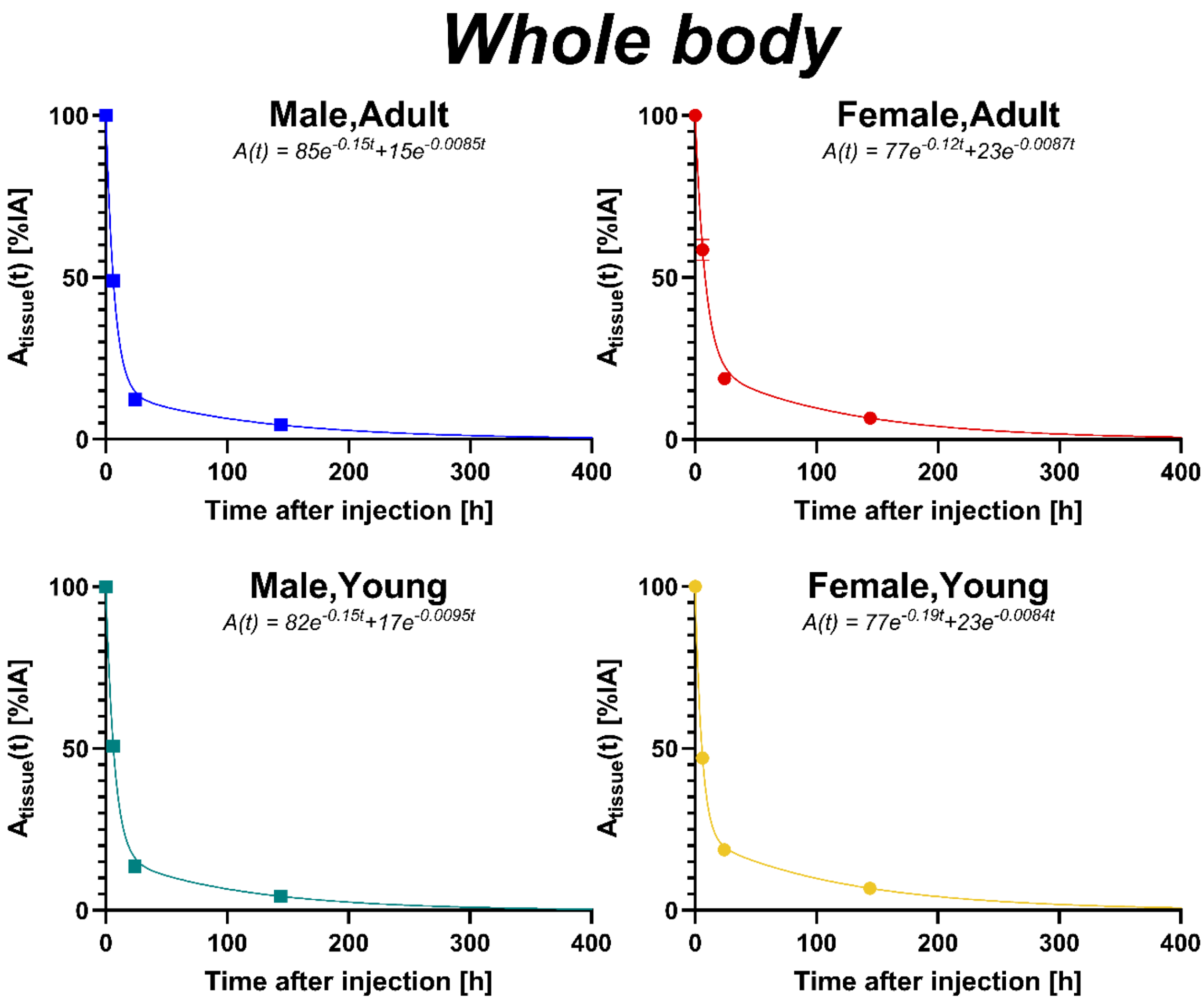

Figure S17: Biokinetic curves for whole-body activity. Summ of the total measured ^{131}I activity, $A_{\text{tissue}}(t)$, together with the fitted biexponential biokinetic curves for male and female Sprague–Dawley rats exposed at 5 (young) or 17 (adult) weeks of age. The fitted equations used to estimate the time-integrated whole-body activity for calculation of the photon contribution to the absorbed dose are shown in each panel. Data are presented as mean ± SEM.
