# Supplementary material for: Sex and age at exposure influence 131I biodistribution and dosimetry in Sprague–Dawley rats
Source: Biol Sex Differ. 2026 Sep 18;17:154. doi: 10.1186/s13293-026-00989-4 (PMC13589867; doi:10.1186/s13293-026-00989-4)
Supplement: Supplementary file 5 [file 13293_2026_989_MOESM7_ESM.pdf]

Additional file 7

Full uncropped western blot membrane images used for NIS protein quantification.

Uncropped western blot membrane images showing Ponceau S staining, glyceraldehyde 3-phosphate dehydrogenase (GAPDH, Cell Signaling Technology, 14C10, 1:1000) loading controls, and sodium-iodide symporter (NIS, Proteintech, 24324-1-AP, 1:200) protein expression in thyroid tissue samples from male and female Sprague–Dawley rats at the age of 5 (young) and 17 (adult) weeks.

Membranes are shown at the exposure times used for quantification. Samples are grouped according to sex and age at exposure and are labeled accordingly.

A) Ponceau S staining

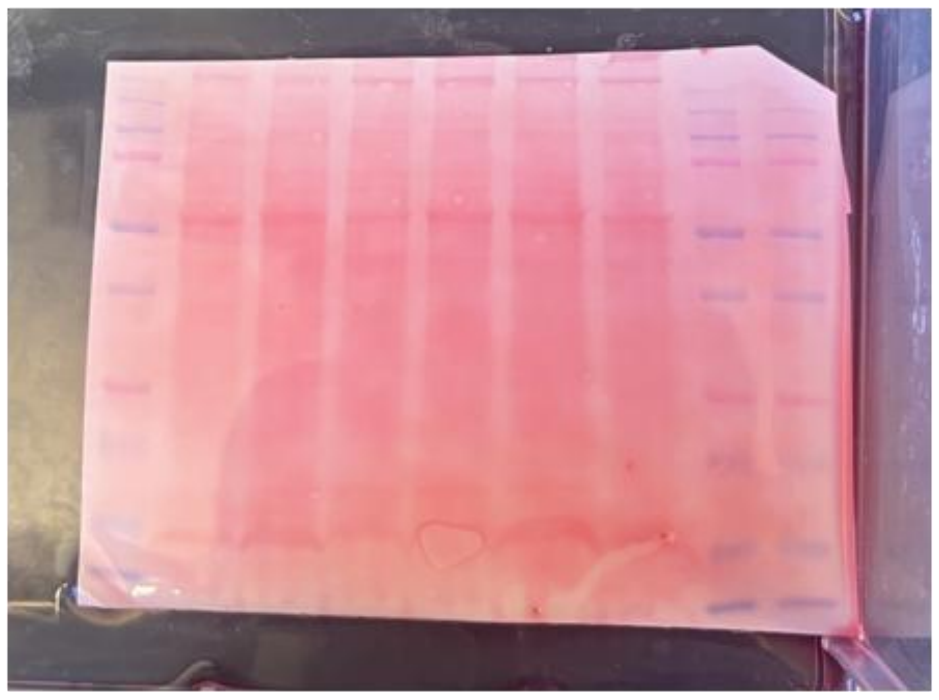

B) GAPDH (500 ms exposure)

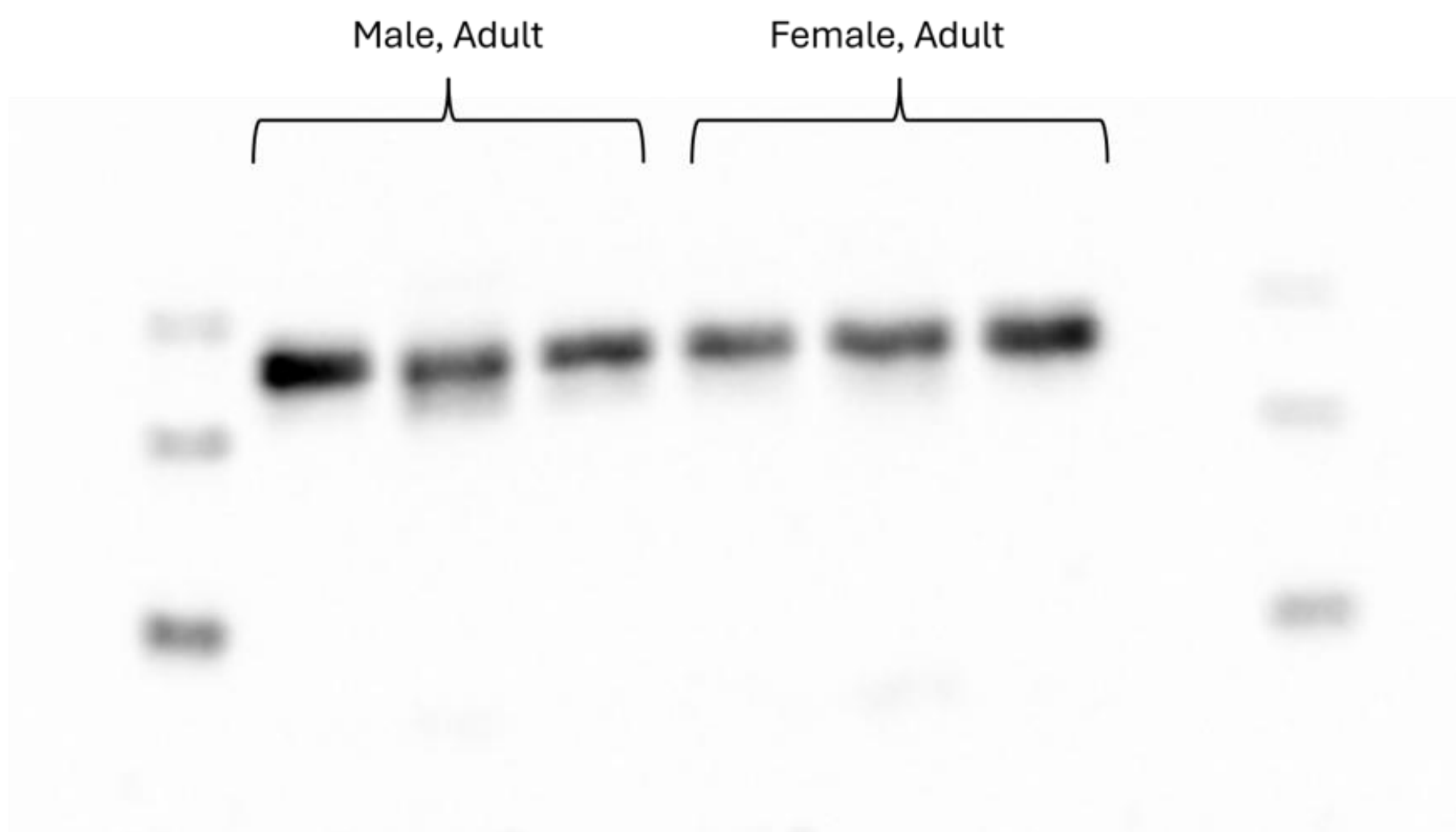

C) NIS (10 s exposure)

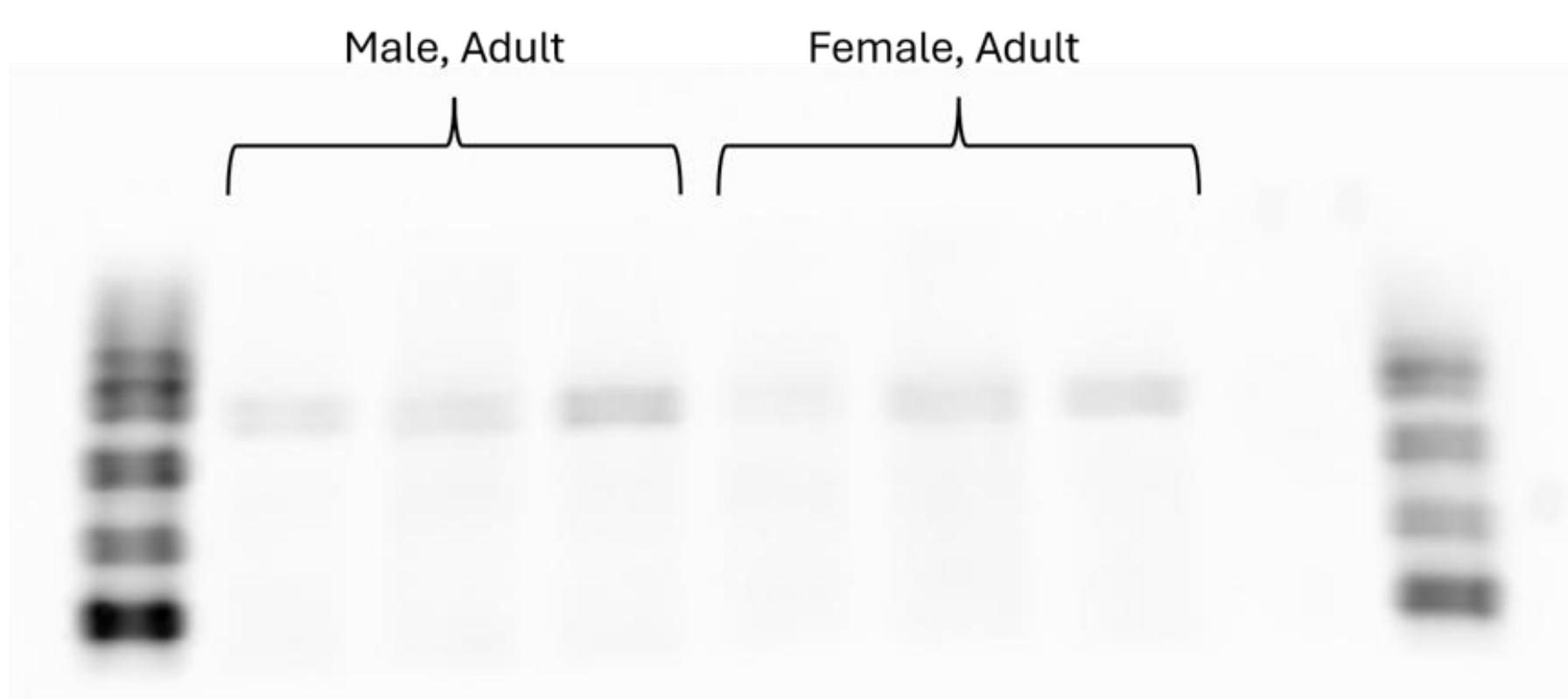

Figure S1: Uncropped western blot membrane images for adult rats. Uncropped membrane images showing **A)** Ponceau S staining, **B)** GAPDH (Cell Signaling Technology, 14C10, 1:1000), and **C)** NIS (Proteintech, 24324-1-AP, 1:200) in thyroid tissue samples from male and female Sprague–Dawley rats exposed at 17 weeks of age.

A) Ponceau S staining

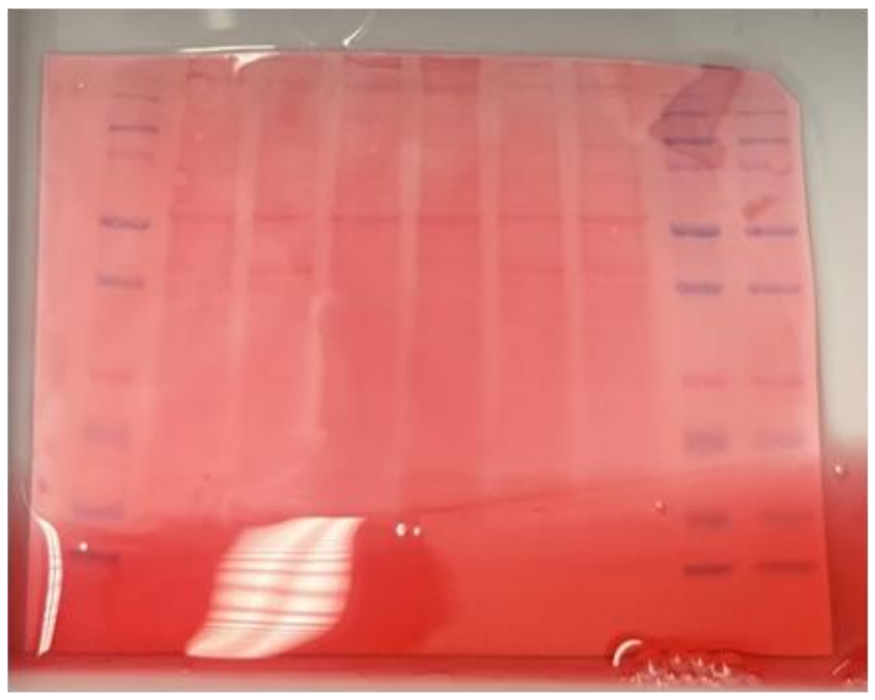

B) GAPDH (100 ms exposure)

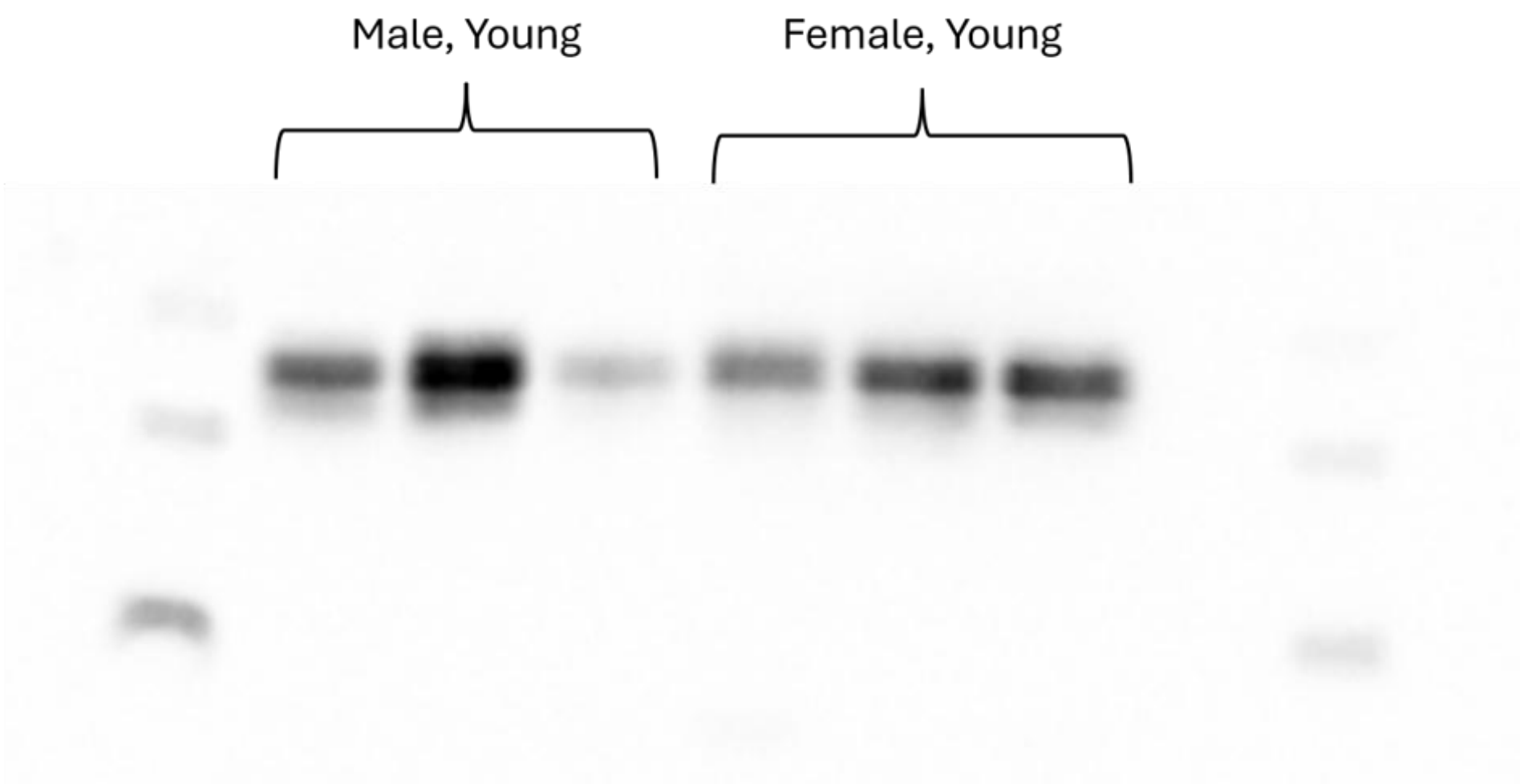

C) NIS (10 s exposure)

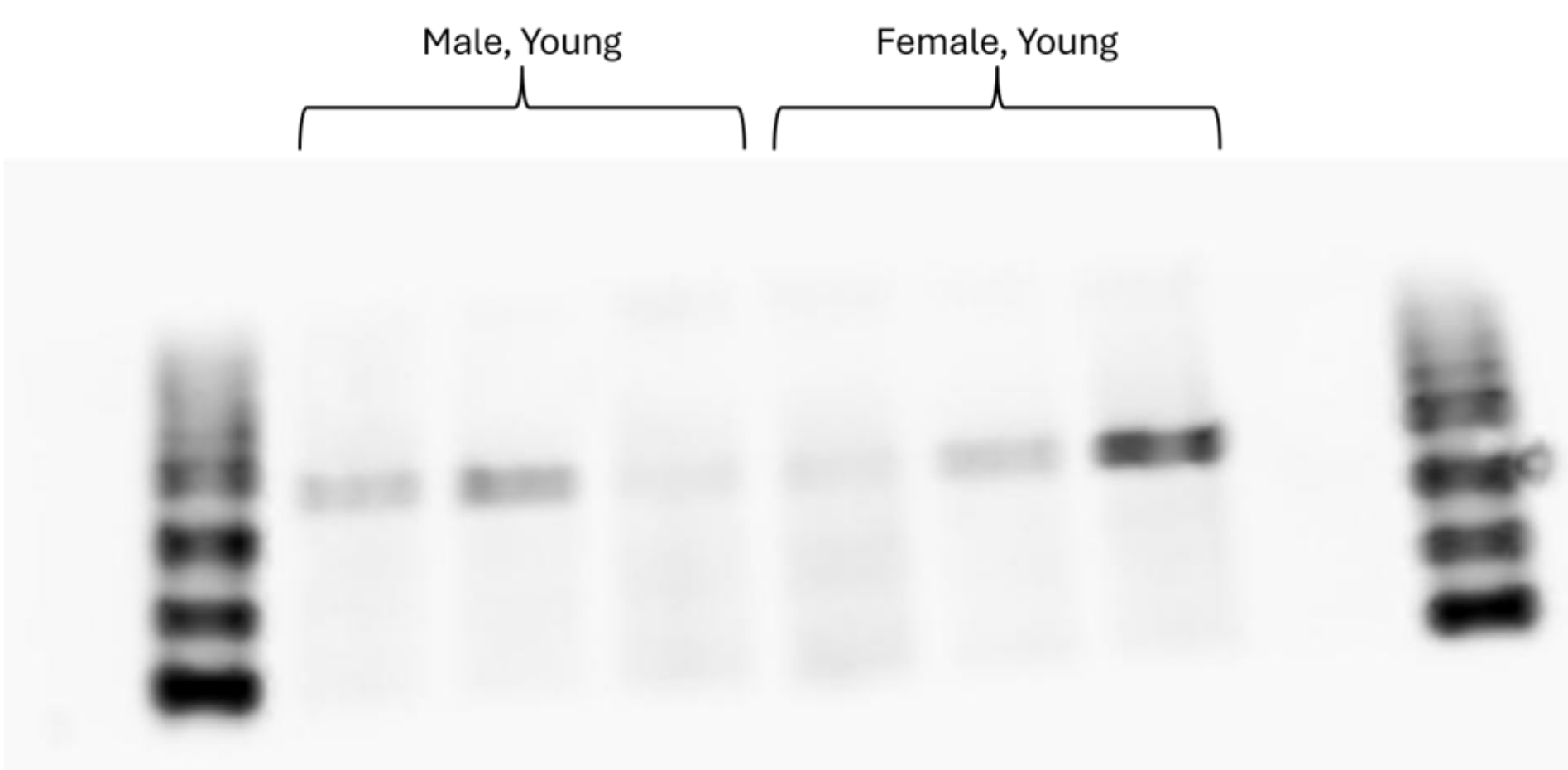

Figure S2: Uncropped western blot membrane images for young rats. Uncropped membrane images showing **A)** Ponceau S staining, **B)** GAPDH (Cell Signaling Technology, 14C10, 1:1000), and **C)** NIS (Proteintech, 24324-1-AP, 1:200) in thyroid tissue samples from male and female Sprague–Dawley rats exposed at 5 weeks of age.
